# Transplantation of bacteriophages from ulcerative colitis patients shifts the gut bacteriome and exacerbates the severity of DSS colitis

**DOI:** 10.1186/s40168-022-01275-2

**Published:** 2022-07-08

**Authors:** Anshul Sinha, Yue Li, Mohammadali Khan Mirzaei, Michael Shamash, Rana Samadfam, Irah L. King, Corinne F. Maurice

**Affiliations:** 1 Department of Microbiology & Immunology, McGill University, Montreal, QC Canada; 2 Department of Endocrinology and Metabolism, Guangdong Provincial Key Laboratory of Diabetology, The Third Affiliated Hospital of Sun Yat-Sen University, Guangzhou, 510630 Guangdong China; 3 Institute of Virology, Helmholtz Center Munich and Technical University of Munich, 85764 Neuherberg, Bavaria Germany; 4 Charles River Laboratories, 22022 Transcanadienne, Senneville, QC H9X 3R3 Canada; 5 McGill Interdisciplinary Initiative in Infection and Immunity, Montreal, QC Canada

**Keywords:** Bacteriophages, Inflammatory bowel disease, DSS colitis, Ulcerative colitis, Microbiota, Intestine

## Abstract

**Background:**

Inflammatory bowel diseases (IBDs) including Crohn’s disease (CD) and ulcerative colitis (UC) are characterized by chronic and debilitating gut inflammation. Altered bacterial communities of the intestine are strongly associated with IBD initiation and progression. The gut virome, which is primarily composed of bacterial viruses (bacteriophages, phages), is thought to be an important factor regulating and shaping microbial communities in the gut. While alterations in the gut virome have been observed in IBD patients, the contribution of these viruses to alterations in the bacterial community and heightened inflammatory responses associated with IBD patients remains largely unknown.

**Results:**

Here, we performed in vivo microbial cross-infection experiments to follow the effects of fecal virus-like particles (VLPs) isolated from UC patients and healthy controls on bacterial diversity and severity of experimental colitis in human microbiota-associated (HMA) mice. Shotgun metagenomics confirmed that several phages were transferred to HMA mice, resulting in treatment-specific alterations in the gut virome. VLPs from healthy and UC patients also shifted gut bacterial diversity of these mice, an effect that was amplified during experimental colitis. VLPs isolated from UC patients specifically altered the relative abundance of several bacterial taxa previously implicated in IBD progression. Additionally, UC VLP administration heightened colitis severity in HMA mice, as indicated by shortened colon length and increased pro-inflammatory cytokine production. Importantly, this effect was dependent on intact VLPs.

**Conclusions:**

Our findings build on recent literature indicating that phages are dynamic regulators of bacterial communities in the gut and implicate the intestinal virome in modulating intestinal inflammation and disease.

Video Abstract

**Supplementary Information:**

The online version contains supplementary material available at 10.1186/s40168-022-01275-2.

## Background

The human gut microbiota is a complex community of microorganisms including bacteria, viruses, archaea, and various eukarya, all of which provide protection against pathogens and maintain metabolic and immunological homeostasis [1, 2]. Intestinal bacterial communities are particularly important in guiding the appropriate development of different immune cell types and regulating the balance between pro- and anti-inflammatory responses in the gut [2–6]. For these reasons, alterations in the gut bacteriome have been associated with various immunological disorders, including inflammatory bowel diseases (IBDs) [7]. IBDs, comprised of Crohn’s disease (CD) and ulcerative colitis (UC), are chronic conditions in which regions of the gut are inflamed and ulcerated, often leading to debilitating abdominal pain, rectal bleeding, and diarrhea. In IBD patients there is often a reduction in bacterial diversity, including a decrease in the proportion of immunoregulatory short-chain fatty acid (SCFA)-producing *Clostridia* and an increase in tissue-invasive *Enterobacteriaceae* [7–10]. Consistent with these clinical results, several of these bacterial taxa have been shown to influence colitis severity in mouse models of intestinal inflammation [11–15]. In addition to these changes in the gut microbiota, population-based genetic studies have revealed that several IBD risk alleles are involved in host-microbe interactions [16, 17]. Together, these observations have led to the general assumption that IBD is the result of an inappropriate intestinal immune response towards the gut microbiota in a genetically susceptible host. Despite our understanding of genetics and changes to the gut microbiota in IBD, the precise factors that drive bacterial alterations in IBD are not well understood.

Bacteriophages (phages), which are viruses that infect bacteria, are present at similar abundances as their bacterial hosts in the human gut and have shown to be strong regulators of bacterial communities in the mammalian gut [18–22]. Recent work has shown that in vivo administration of phages in mice can alter bacterial diversity [21–24], disrupt bacterial interaction networks [19], and alter the concentration of bacterial-derived metabolites [19]. Importantly, there is emerging data to support the idea that phages can alter disease outcomes by regulating the composition and diversity of their bacterial hosts in the gut [21, 24, 25].

Given the limitations of culturing gut bacteria and their associated phages, virome characterization has primarily relied on metagenomic sequencing of fecal or gut mucosal samples. However, due to the extensive diversity of phages and their low representation in databases, it has been challenging to link phages to their bacterial hosts or gain taxonomic information from viral sequence data alone [26]. Still, recent improvements in gut virome databases [27, 28] and bioinformatic tools for detecting viruses [29] have revealed some consistent characteristics of human gut viromes. Specifically, phage communities from healthy adults are unique [30], stable over time [30, 31], and dominated by dsDNA *Caudovirales* phages and ssDNA *Microviridae* phages [30, 32–34]. Compared to other ecosystems, there also tends to be low virus-to-bacteria ratios (VBRs) [20], a high proportion of bacteria containing predicted prophages [35], and a high prevalence of ubiquitous crAss-like phages shown to infect *Bacteroides* in gut virome samples [28, 30, 36].

Accumulating evidence from metagenomic sequencing of fecal and mucosal samples indicates that the gut virome is altered in IBD patients, whether in CD or UC cohorts, and adults or children [37–40]. Several of these reports have shown increases in the abundance and richness of the order *Caudovirales* in IBD patients [37–39]. Some also report an increase in the relative abundance of phages predicted to infect Firmicutes, which are typically reduced in IBD and contain several species that induce anti-inflammatory immune responses [37, 41]. Most recently, Cloone*y* et al. [37] showed that, in addition to shifts in viral diversity, there was also an increased relative abundance of phages classified as temperate in UC and CD patients. These observations suggest that inflammatory events may initiate prophage induction in gut bacteria and a switch from the lysogenic replication cycle to lytic replication [37]. Similar observations in mouse models of colitis further support a link between intestinal inflammation and alterations of the gut virome [42].

These data collectively support a relationship between IBD and intestinal phage communities. However, whether phage alterations impact gut bacterial communities, intestinal immune responses, and/or disease progression is unknown. Here, we investigated the effects of administering fecal virus-like particles (VLPs) from UC patients (UC VLPs) and non-IBD controls (healthy VLPs) to human microbiota-associated (HMA) mice, and their subsequent impact on the gut microbiome and dextran sodium sulfate (DSS)-induced colitis.

## Results

### Experimental model and composition of pooled viral and bacterial inoculums

**Fig. 1:**
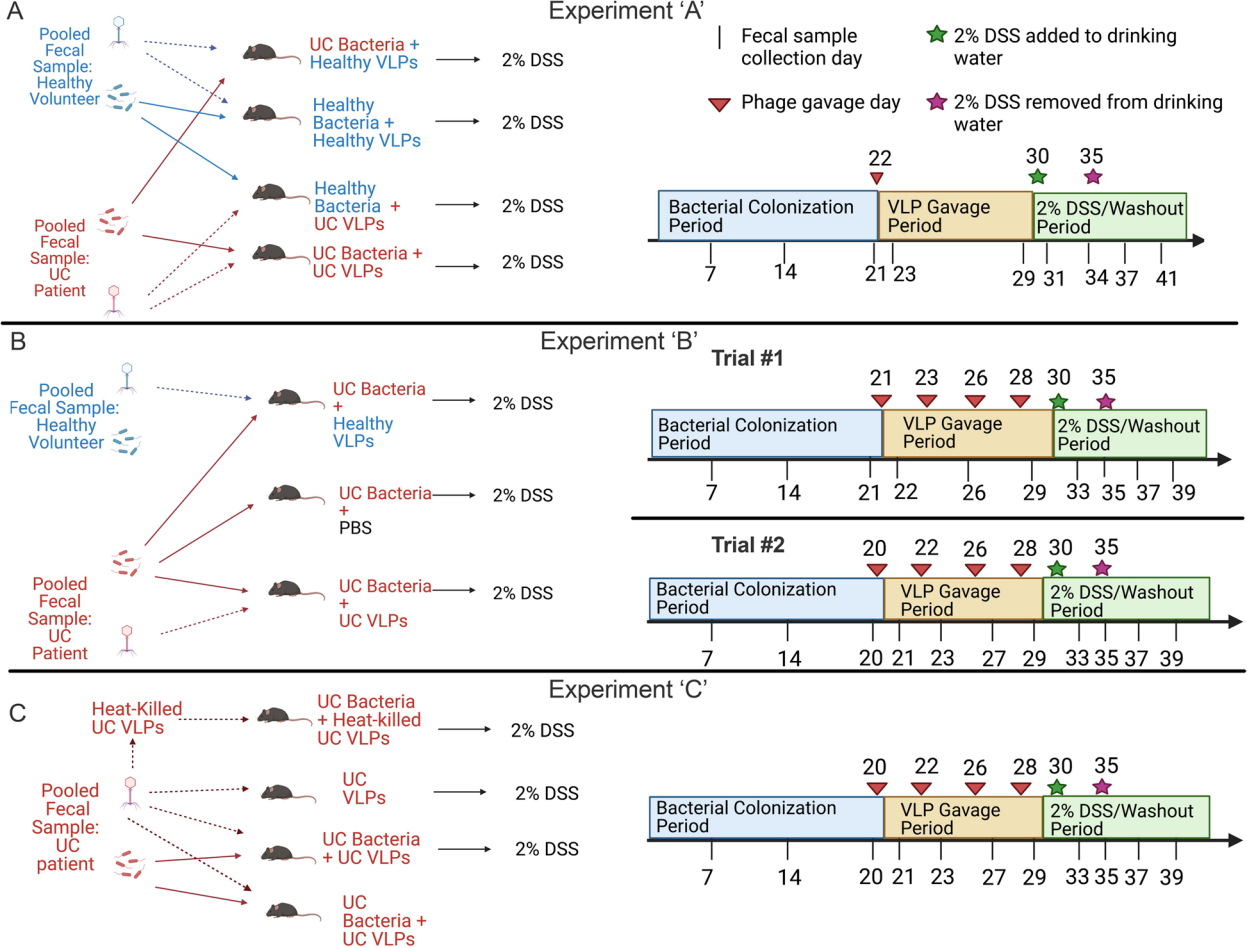
Schematic and timeline of the experimental model. **A** HMA mice administered healthy or UC bacterial communities were given a single dose of healthy or UC VLPs, followed by 2% DSS. **B** UC-HMA mice were given four doses of healthy VLPs, UC VLPs, or PBS, followed by 2% DSS. **C** UC-HMA or GF mice were given four doses of UC VLPs or heat-killed UC VLPs. All groups were then given 2% DSS, except one group of UC-HMA mice given UC VLPs. Each treatment group included 5 or 6 GF or HMA mice per experiment housed in three separate cages. In each experiment 200 μL of bacterial and VLP communities were administered to mice by oral gavage at equal concentrations (1–3 × 10^8^ VLPs or bacterial cells/mL)

In order to determine the effects of healthy and UC VLPs on bacterial and DSS colitis severity, we performed in vivo “cross-infection” experiments in HMA mice (Experiments “A–C,” outlined in Fig. 1). Germ-free (GF) mice were first colonized with pooled bacterial communities from 3 healthy volunteers or 3 UC patients (healthy-HMA mice, UC-HMA mice). Following bacterial colonization, mice were given single or multiple doses of VLPs, followed by 2% DSS (Fig. 1). The use of 2% DSS to induce colitis allowed for the temporal control of mild inflammation, thus enabling us to study VLP-mediated effects on bacterial communities both independent of, and in the presence of, intestinal inflammation. In addition, as DSS-induced inflammation is largely restricted to the colon, our model mimics pathology similar to that observed in UC patients [43].

**Fig. 2:**
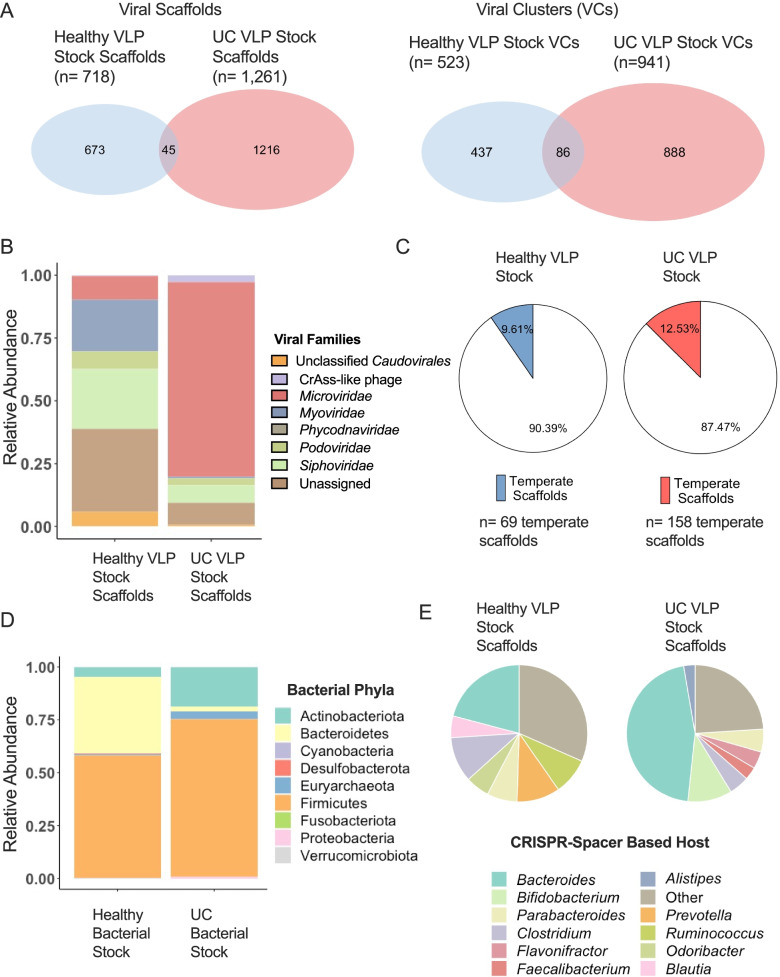
Composition of pooled healthy and UC VLP and bacterial inoculums. **A** Shared and unique viral scaffolds and VCs between pooled healthy and UC VLP inoculums. **B** Relative abundance of viral families in each VLP inoculum. **C** Proportion of scaffolds in VLP inoculums identified as temperate. **D** Relative abundance of bacterial phyla in each pooled bacterial inoculum. **E** Proportion of scaffolds based on CRISPR spacer predicted hosts. The top 7 most prevalent host predictions in each treatment group are displayed. VLP shotgun metagenomics was used for VLP inoculum analyses and 16S rRNA gene sequencing of the V4 region was used for bacterial inoculum analyses. Inoculum samples were pooled using equal weight of 3 fecal samples from healthy volunteers or UC patients

To first characterize the virome of the pooled healthy and UC VLP inoculums given to HMA mice, we performed shotgun sequencing on VLP fractions from fecal samples. Quality-filtered reads from each sample were assembled into scaffolds, and viral scaffolds were detected and annotated (see the “Methods” section, Supplementary Fig. S1). Of the 2063 assembled scaffolds greater than 3 kb in length assembled from healthy and UC inoculum samples, 1791 (86.8%) were classified as viral using our methodology. In total, 718 and 1261 viral scaffolds were found in the pooled healthy and UC VLP inoculums, respectively (Fig. 2A, Supplementary Table S1). In agreement with the reported high inter-individuality of virome samples and differences in virome composition between disease states [30, 37], only 45 scaffolds were shared between these inoculums (Fig. 2A). We also used vConTACT2 to form viral clusters (VCs) based on shared protein-coding genetic content in order to account for the high-inter individuality of these viromes [44]. Using this approach, 523 and 941 VCs (including singletons) were found in the healthy VLP and UC VLP inoculums, respectively (Fig. 2A, Supplementary Table S1). Of these VCs, only 86 were shared between the two pooled inoculums (Fig. 2A), suggesting that a substantial portion of these viral inoculums remained unique at this high taxonomic level. Using a vote-based approach to assign viral taxonomy to the VLP scaffolds [30], we also observed differences in the viral families present in each inoculum (Fig. 2B). The healthy inoculum virome was predominately composed of dsDNA phages belonging to the order *Caudovirales* and the families *Myoviridae* and *Siphoviridae* and unclassified viral scaffolds (Fig. 2B)*.* In contrast, the pooled UC inoculum virome was dominated (77.4% relative abundance) by scaffolds belonging to the (ssDNA) *Microviridae* family, along with crAss-like phages and phages belonging to the *Siphoviridae* and *Podoviridae* families (Fig. 2B). These data are consistent with previous studies, showing that individual gut viromes can be dominated by ssDNA *Microviridae* [30, 32, 34, 37]. Using the Random Forest Classifier, BACPHLIP, we were also able to classify viral scaffolds in our dataset as temperate [45]. The pooled UC inoculum contained both higher absolute numbers of unique scaffolds identified as temperate and a higher proportion of temperate scaffolds (Fig. 2C, Supplementary Table S1), in line with previous associations between temperate phages and IBD [37].

In addition to these differences in virome composition, we also used 16S rRNA gene sequencing of the V4 region to determine the composition of the pooled healthy and UC bacterial inoculums used to colonize GF mice. Phylum-level analysis revealed decreased relative abundance of Bacteroidetes (2.13% UC, 36.32% healthy), increased Actinobacteria (18.80% UC, 4.63% healthy), and increased Proteobacteria (0.80% UC, 0.15% healthy) in the pooled UC inoculum (Fig. 2D, Supplementary Table S1), consistent with previous observations of UC bacterial communities [46–48]. We also compared genus-level differences between the bacterial inoculums with differences in genus-level bacterial host predictions of VLP scaffolds in our dataset using clustered regularly interspaced short palindromic repeats (CRISPR) spacer homology [49]. In total, 196/718 (27.92%) of healthy VLP inoculum scaffolds were successfully assigned CRISPR spacer-based genus predictions and 296/1261 (23.47%) of UC VLP inoculum scaffolds were assigned genus-level predictions (Supplementary Table S1). Some genus-level differences in relative abundance in the pooled bacterial inoculum were consistent with differences in genus-level bacterial host predictions of VLP scaffolds, indicative of concordance between the viral and bacterial fractions of these inoculums. For instance, increased relative abundance of *Bifidobacterium* in the UC bacterial inoculums (9.87% UC, 1.38% healthy) was consistent with a higher percentage of VLP scaffolds predicted to infect *Bifidobacterium* (10.47% UC, 3.57% healthy) (Fig. 2E, Supplementary Table S1)*.* Additionally, *Prevotella* was present at high relative abundances (21.10%) in the healthy bacterial inoculum and was not detected in the UC bacterial inoculum (Supplementary Table S1). This disparity in *Prevotella* abundance was reflected in a high proportion of *Prevotella*-infecting VLPs in the healthy inoculum (10.20%) and zero scaffolds in the UC VLP inoculum predicted to infect *Prevotella* (Fig. 2E, Supplementary Table S1). Interestingly, *Bacteroides*-infecting VLPs made up 45.61% of UC VLP inoculum scaffolds with predicted CRISPR spacer hosts (Fig. 2E, Supplementary Table S1), despite low *Bacteroides* relative abundance (1.79%) in the UC bacterial inoculum (Supplementary Table S1), which could be reflective of an expansion of phages targeting and depleting *Bacteroides* in these UC patients. Together, our data highlight UC-specific alterations in both the viral and bacterial fractions of the pooled fecal samples used for VLP cross-infection experiments in HMA mice.

### UC bacterial communities enhance DSS colitis severity in comparison to bacterial communities from healthy controls

To first determine the effects of single healthy and UC VLPs doses on bacterial diversity and DSS colitis severity, we colonized GF mice with the pooled healthy and UC bacterial communities described above (Fig. 2D). After 21 days of bacterial colonization, mice were given a single dose of either healthy or UC VLPs, followed by 2% DSS on day 30 (Fig. 1A). After a single dose of VLPs, we did not observe significant differences in bacterial beta-diversity by weighted UniFrac distance between mice given healthy or UC VLPs in either healthy or UC-HMA mice at any sampling point (Supplementary Tables S2 and S3). Using ANCOM II, a statistical framework that accounts for the underlying structure of microbial communities [50], we were also unable to identify any species that were differentially abundant between mice given healthy and UC VLPs in healthy or UC-HMA mice during the VLP gavage period and DSS/washout periods. Similarly, there were no significant differences in DSS colitis severity in HMA mice given healthy or UC VLPs (Supplementary Fig. S2), suggesting that single doses of VLPs did not alter bacterial community composition or regulate intestinal inflammation.

**Fig. 3:**
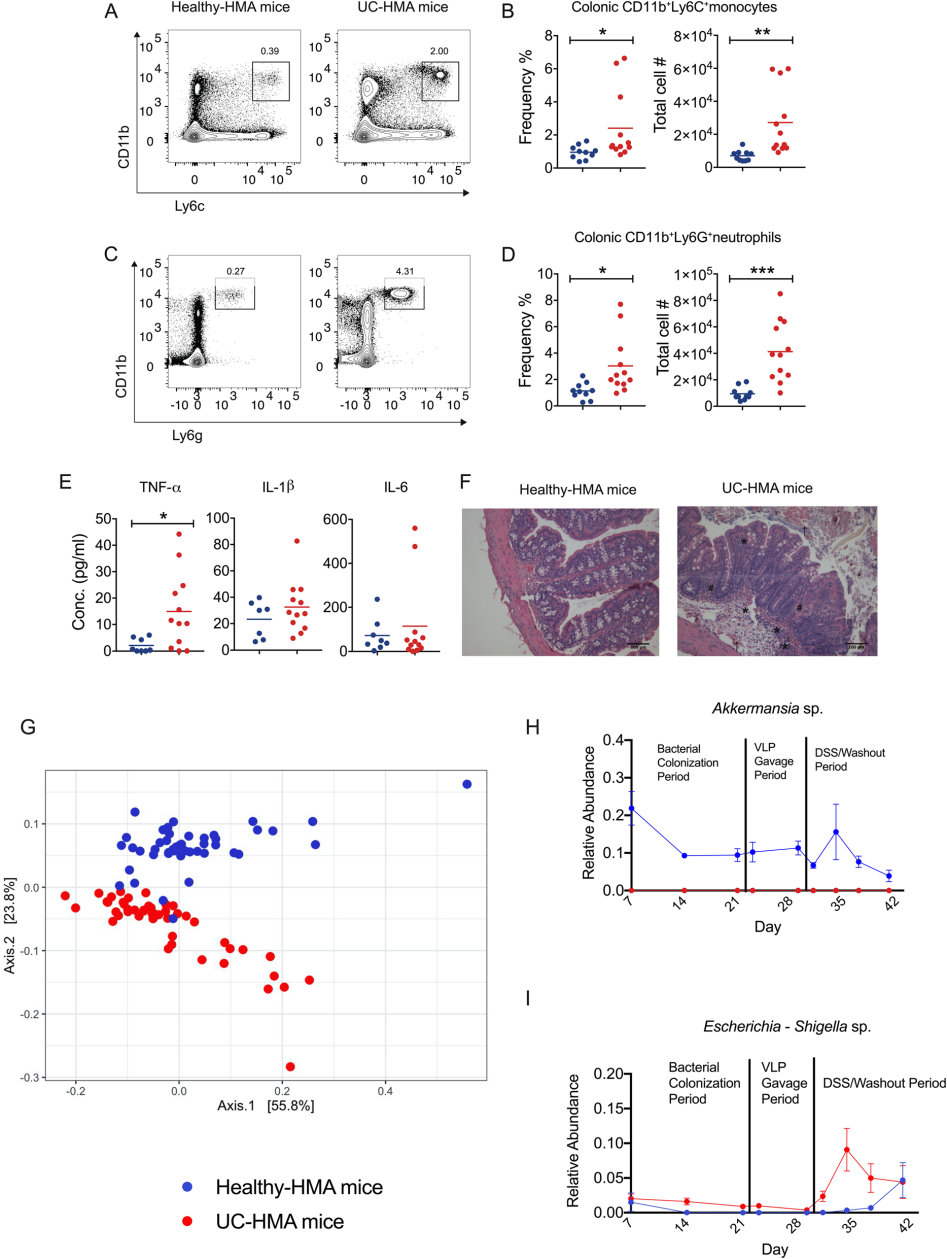
Mice colonized with UC patient-derived fecal bacteria exhibit increased inflammation during experimental colitis. Data shown is from experiment “A.” **A** Representative contour plots and **B** mean frequency and absolute number of colonic inflammatory monocytes (CD11b^+^Ly6C^+^Ly6G^−^) at day 10 post-DSS administration. **C** Representative contour plots and **D** mean frequency and absolute number of colonic neutrophils (CD11b^+^Ly6C^−^Ly6G^+^) at day 10 post-DSS administration. **E** Mean TNF-α, IL-1β, and IL-6 production from colon tissue explants at day 10 post-DSS administration. **F** Representative H&E staining of paraffin-embedded cross colon sections at day 10 post-DSS administration (scale bars are 100μm). Asterisk (*) indicates area of cellular infiltration; number sign (#) indicates area of distorted crypt architecture; black arrow indicates the area of bleeding. Data were analyzed using a two-tailed unpaired parametric *t* test (*p < 0.05, **p < 0.01, ***p < 0.001). **G**–**I** 16S rRNA gene sequencing of HMA mouse fecal bacteria. **G** PCoA on weighted UniFrac distances. **H**, **I** Mean relative abundance of *Akkermansia* sp*.* (**H**) and *Escherichia-Shigella* sp. (**I**) over time in HMA mice. Species were confirmed to be differentially abundant using ANCOM II. Error bars, SE. **B**, **D**, **E** Error bars, SD. Data shown from one experiment. Dots in **B**, **D**, and **E** represent individual mice (*n*=8 healthy-HMA mice, *n*=12 UC-HMA mice). Dots in **G**, **H**, and **I** indicate pooled mouse fecal samples at a single sampling point. At each sampling point, mouse fecal samples in each cage were pooled from 1 or 2 mice (*n*=6 cages per group, 1 or 2 mice per cage)

However, regardless of whether healthy or UC VLPs were administered, UC-HMA mice had increased colitis severity compared to healthy-HMA mice as determined by innate immune cellular infiltration, inflammatory cytokine secretion in colonic explants, and tissue histology (Fig. 3A–F). Overall, these data are consistent with previous studies [51, 52] indicating that gut bacteria from UC patients predisposes HMA mice to an enhanced form of colitis. To determine the differences between the gut bacterial communities in mice humanized with microbial communities from UC patients or healthy volunteers, we performed 16S rRNA gene sequencing on mouse fecal pellets. Principal coordinate analysis (PCoA) on weighted UniFrac distances including all sampling points revealed differences in bacterial beta-diversity between healthy and UC-HMA mice (Fig. 3G). PERMANOVA analyzes revealed significant differences in beta-diversity between these groups at 8/9 of the sampling points sampled (Supplementary Table S4). We next tested for treatment-specific differences in bacterial species previously associated with human IBD or experimental colitis severity. Using ANCOM II, we identified 68/142 species that were differentially abundant between mice given bacterial communities from healthy volunteers and UC patients (ANCOM cutoff W > 0.6, Supplementary Table S5). HMA mice humanized with UC bacteria showed reduced proportions of *Akkermansia* sp. across all sampling points (Fig. 3H), a bacterial genus typically reduced in IBD patients and shown to ameliorate DSS colitis [12, 53]. It is also well established that bacteria from the *Enterobacteriaceae* family increase in abundance in IBD and exacerbate experimental colitis severity [13, 14]. Accordingly, we found an expansion of *Escherichia*-*Shigella* sp. during DSS colitis*,* only in mice humanized with UC bacteria (Fig. 3I). Importantly, in the bacterial inoculums used to gavage the HMA mice, there were similar increases in *Escherichia*-*Shigella* sp. (0.45% UC, 0.069% healthy) and decreases in *Akkermansia* sp*.* (0% UC, 2.14% healthy) (Supplementary Table S1) in the UC samples compared to healthy volunteers. Together, these colonization-specific differences in bacterial taxa may explain the exacerbation of DSS colitis in UC-HMA mice.

### Administration of healthy and UC VLPs increases fecal viral abundance and virus-to-bacteria ratio (VBR) in HMA mice

We next wanted to determine whether multiple VLP doses from healthy volunteers could alter the gut bacteriome and prevent the exacerbation of DSS colitis. As we did not observe noticeable changes in bacterial community composition (Supplementary Tables S2 and S3) or DSS colitis severity (Supplementary Fig. S2) following a single dose of healthy or UC VLPs, we explored whether multiple doses of healthy or UC VLPs would alter the gut microbiota of UC-HMA mice (Fig. 1B, C). One common approach in phage therapy to increase treatment efficacy is to add multiples doses instead of one single dose to sustain high phage densities [54]. Thus, for the remaining experiments, we proceeded to give HMA mice multiple repeated doses of VLPs.

**Fig. 4:**
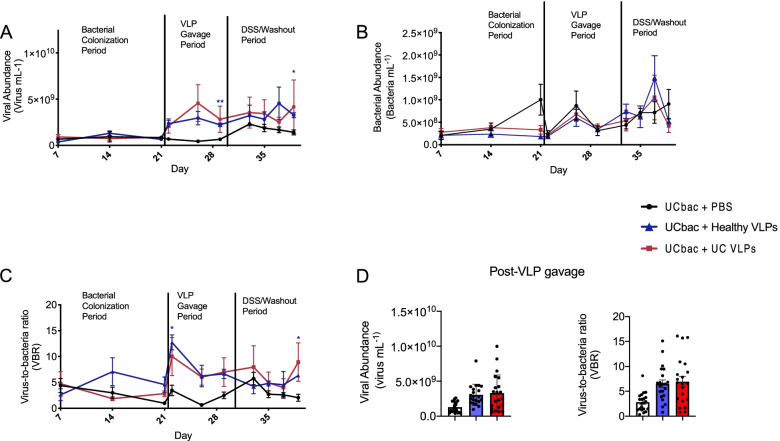
Healthy and UC VLP administration increases viral abundance and VBR in UC-HMA mice. Data shown is from experiment “B” (trial #1). **A** Viral abundance was determined from mouse fecal pellets using epifluorescence microscopy and compared to **B** bacterial abundances obtained by flow cytometry after staining with SybrGREEN I to obtain **C** VBRs. **D** Mean total viral abundance and mean total VBR post-VLP gavage were compared between treatment groups after the first dose of VLPs or PBS was given to mice. **A**–**C** Significance was assessed using a repeated measure two-way ANOVA and Dunnett’s multiple comparisons test (*p ≤ 0.05, **p≤ 0.01) and using a Geisser-Greenhouse correction. Red and blue asterisks indicate significant differences between the PBS control and HMA mice given UC VLPs and healthy VLPs, respectively. Dots represent abundance or VBR of pooled mouse fecal samples at a single sampling point. At each sampling point, mouse fecal samples in each cage were pooled from 2 mice (*n*=3 cages per group, 6 mice per group). Error bars, SE. UC bac, UC-HMA mice

In the first experiment (experiment “B,” Fig. 1B), UC-HMA mice were given four doses of healthy VLPs, UC VLPs, or PBS over the course of 9–10 days. Following the VLP gavage period, mice were given 2% DSS followed by a washout period. This first experiment was performed independently twice (trial #1, trial #2). In a second experiment (experiment “C,” Fig. 1C), we tested the impact of phage viability as well as bacteria-independent effects of multiple doses of VLPs on intestinal inflammation using UC-HMA mice administered intact or heat-killed UC VLPs and GF mice given UC VLPs alone (experiment “C,” Fig. 1C). We first determined whether repeated dosing of VLPs would increase viral abundance and virus-to-bacteria ratio (VBR) in HMA mice. Low levels of VLPs (8.12 × 10^8^ virus mL^−1^) could be detected in HMA mice during the bacterial colonization period (Fig. 4A; experiment “B,” trial #1) before the addition of inoculum VLPs, likely a result of prophage induction of newly colonized bacteria and/or VLPs that we were unable to remove during the separation of the bacterial and VLP fractions in the inoculums. Still, these levels of VLPs during the bacterial colonization period were lower compared to after VLP gavage (3.93-fold increase in UC VLP treated mice and 3.91-fold increase in healthy VLP treated mice) (Fig. 4A). Additionally, regardless of the source (healthy or UC samples), repeating VLP dosing generally increased viral abundance and virus-to-bacteria ratio (VBR) during the VLP gavage period relative to PBS (Fig. 4A–D, Supplementary Fig. 3A–D) or heat-killed UC VLP controls (Supplementary Fig. 4A–D).

Despite the general increase in viral abundance and VBR post-VLP gavage, there was no associated decrease in total bacterial abundance compared to PBS (Fig. 4B, Supplementary Fig. S3B) or to heat-killed controls (Supplementary Fig. S4B), which suggests that bacteria killed by phage-mediated lysis may be rapidly replaced by genetically or phenotypically resistant bacterial taxa [19, 55]. Notably, there was no detectable increase in viral abundance in GF mice given UC VLPs alone, suggesting that phage persistence required bacterial hosts (Supplementary Fig. S4A).

Since some ecological models predict that more metabolically active bacteria are less susceptible to lytic phage infection [56], we next determined if healthy and UC VLPs could alter the proportion of active bacterial cells. Using SYBRGreen I staining and flow cytometry, we determined the proportion of metabolically active fecal bacteria in HMA mice, as described previously [57] (Supplementary Fig. S5A). While not necessarily specific to measuring bacterial growth rate, SYBRGreen I staining is commonly used as a broad marker for bacterial metabolic activity [57, 58]. Compared to PBS and heat-killed UC VLP controls, healthy and UC VLPs did not drastically shift the proportion of active bacteria (Supplementary Fig. S5B–D), except at one sampling point where the proportion of active cells significantly increased in both groups of mice given UC VLPs compared to the heat-killed UC VLP control (Supplementary Fig. S5D). We also used Propidium Iodide (PI) staining to determine the proportion of damaged bacterial cells in HMA mice (Supplementary Fig. S6A). Healthy and UC VLPs also did not dramatically shift the proportion of damaged cells compared to PBS or heat-killed controls (Supplementary Fig. S6B–D), with the exception of a single time-point, where UC VLPs decreased the proportion of damaged bacteria (Supplementary Fig. S6C). Together, these data suggest that donor VLPs can interact with HMA mice but are not able to shift the proportions of active or damaged gut bacterial cells.

### Fecal VLPs derived from healthy volunteers and UC patients drive unique changes in the virome of HMA mice

Shotgun sequencing was also performed on the VLP fraction of mouse fecal pellets in experiment “B” (trial #1) to follow changes in virome composition of HMA mice following healthy or UC VLP gavage. Of the 6742 scaffolds greater than 3 kb in length assembled from mouse samples, 4219 (62.6%) were identified as viral using our methodology (Supplementary Fig. S1). To assess the effectiveness of the transfer of VLPs from the pooled inoculums to the HMA mice, we determined the proportion of scaffolds found in the VLP inoculums that were only found in HMA mice post-VLP gavage. In total, 76/718 (10.58%) of healthy VLP inoculum scaffolds were also found in HMA mice post-healthy VLP gavage and not pre-VLP gavage (Supplementary Table S6). In contrast, a higher number and proportion of UC VLP inoculum scaffolds, reaching 203/1261 (16.10%), were found in HMA mice post-UC VLP gavage and not found pre-VLP gavage, possibly reflective of autologous transfer of UC VLPs to UC-HMA mice (Supplementary Table S6). In line with these data, post-VLP gavage, we observed increased richness at the scaffold and VC level in HMA-mice given healthy and UC VLPs compared to the PBS control (Supplementary Fig. S7). To further assess the effectiveness of VLP transfer, we measured the Jaccard distance of VCs between each inoculum and HMA samples before and after transfer to HMA mice. While not significant, there was a reduction in Jaccard distance between the viromes of pooled healthy inoculum and the UC-HMA mice given healthy VLPs, and between the pooled UC VLP inoculum and UC-HMA mice given UC VLPs (Supplementary Fig. S8), suggesting increased virome similarity to the viral inoculum over time.

**Fig. 5:**
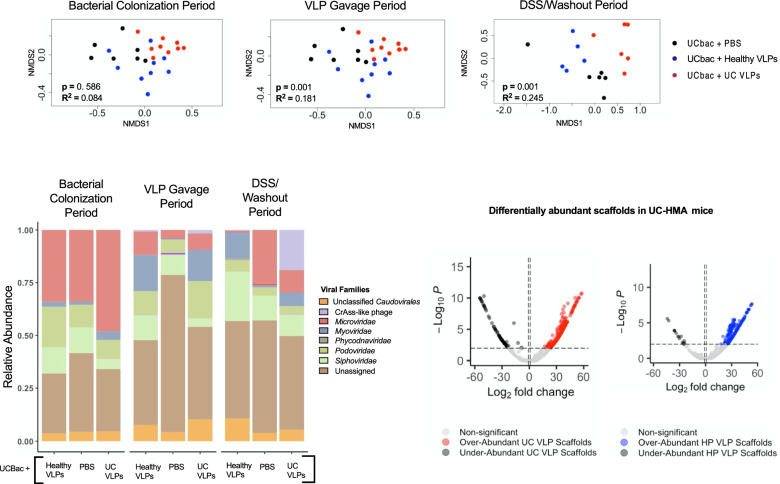
Differences in virome composition of UC-HMA mice after VLP gavage. Data shown is from experiment B (trial #1). **A** NMDS of Bray-Curtis dissimilarity of scaffolds between HMA mice given healthy VLPs, UC VLPs, or PBS during the (left) bacterial colonization period, (middle) VLP gavage period, or (right) DSS/washout period. Significant differences in Bray-Curtis dissimilarity were assessed in each time period using adonis PERMANOVA (*p* ≤ 0.05). Dots represent pooled mouse fecal samples at a single sampling point. All sampling points of the longitudinal study were included in the NMDS and comparative analyses. NMDS stress: bacterial colonization period (stress = 0.146), VLP gavage period (stress = 0.181), DSS/washout (stress = 0.148). **B** Relative abundance of viral families between treatment groups over the experimental time periods. (C) DESeq2 differentially abundant scaffolds between UC-HMA mice given healthy (left) or UC (right) VLPs compared to the PBS control. Differentially abundant scaffolds represent those that significantly changed after VLP gavage between treatment groups. Scaffolds with adjusted *p* values ≤ 0.01 and with log_2_fold changes greater or less than 1 were considered differentially abundant using a two-tailed wald test. Black points on volcano plots indicate overlayed under-abundant scaffolds. At each sampling point, mouse fecal samples in each cage were pooled from 2 mice (*n*=3 cages per group, 6 mice per group; UCbac, UC-HMA mice)

We next performed NMDS on Bray-Curtis dissimilarity of viral scaffolds and VCs to follow differences in viral beta-diversity over time in UC-HMA mice. Grouping sampling points together within each time period, there were no significant differences in Bray-Curtis dissimilarity during the bacterial colonization period at the scaffold or VC level by PERMANOVA (Fig. 5A, Supplementary Fig. S9). However, after VLP gavage, VLP treatment had a significant effect on the viral scaffold and VC diversity, further indicating successful transfer and replication of donor VLPs (Fig. 5A, Supplementary Fig. S9). Interestingly, the effect size of VLP treatment was greatest during the DSS/washout period, suggesting heightened virome divergence after inflammation (Fig. 5A, Supplementary Fig. S9). In agreement with these data, VLP treatment had a significant effect on Bray-Curtis dissimilarity by PERMANOVA, at each sampling point following VLP gavage (Supplementary Table S7). It should also be noted that there was a close to significant difference in Bray-Curtis dissimilarity before VLP gavage (viral scaffolds: *p* = 0.053, viral clusters: *p* = 0.064), which could indicate that isolator effects during viral colonization may have influenced the virome composition in these mice (Supplementary Table S7).

At the viral family level, following VLP gavage, there was an expansion of *Myoviridae* in mice given healthy (7.20-fold increase) and UC VLPs (3.80-fold increase), which persisted after DSS exposure (Fig. 5B). Interestingly, we also observed a large increase in crAss-like phage relative abundance (21.61-fold) in the DSS/washout period only in mice given UC VLPs (Fig. 5B). To further assess differences between VLP-treated mice and PBS controls, we used DESeq2 to determine viral scaffolds that significantly changed after VLP gavage between treatment groups, while controlling for repeated sampling [59]. In total, there were 332 differentially abundant viral scaffolds between UC VLP-treated mice controls and PBS controls (260 over-abundant, 72 under-abundant) and 262 differentially abundant scaffolds between healthy VLP-treated mice and PBS controls (250 over-abundant, 12 under-abundant). Of these significantly upregulated scaffolds, 50/260 in UC VLP-treated mice and 18/250 in healthy VLP-treated mice were also found in their respective pooled inoculums. Of all the over-abundant scaffolds, mice given healthy or UC VLPs were both enriched with phages predicted to infect *Bacteroides* and *Eubacterium* phages (Supplementary Table S8). However, only UC VLP-treated mice were enriched with crAss-like phages and phages predicted to infect *Erysipelatoclostridium* (Supplementary Table S8). In contrast, healthy VLP-treated mice were enriched with *Myoviridae* phages and phages predicted to infect *Parabacteroides* (Supplementary Table S8). Together, these data indicate that VLPs were transferred from the pooled inoculums to HMA mice, with distinct changes in viral beta-diversity, phage families, and potential phage-bacterial interactions following VLP gavage.

### VLPs derived from healthy individuals or UC patients have distinct effects on the UC gut bacterial communities in vivo

In order to identify whether multiple healthy and UC VLPs doses had distinct effects on bacterial community composition, we performed 16S rRNA gene sequencing on mouse fecal pellets before and after VLP administration, as well as during the DSS/washout period. Using ANCOM II, we identified bacterial species differentially abundant between UC-HMA mice given multiple doses of either UC VLPs, healthy VLPs, or PBS (experiment “B,” summarized in Supplementary Tables S9 and S10). To account for variation due to isolator-specific differences during colonization, bacterial species that were differentially abundant during the baseline bacterial colonization period were not included. In addition, the remaining differentially abundant species were each ranked based on the likelihood that treatment-specific differences were due to VLP treatment or isolator effect (1: likely due to VLP treatment, 2: possibly due to VLP treatment, 3: likely due to isolator effect, see methods for ranking criteria).

**Fig. 6:**
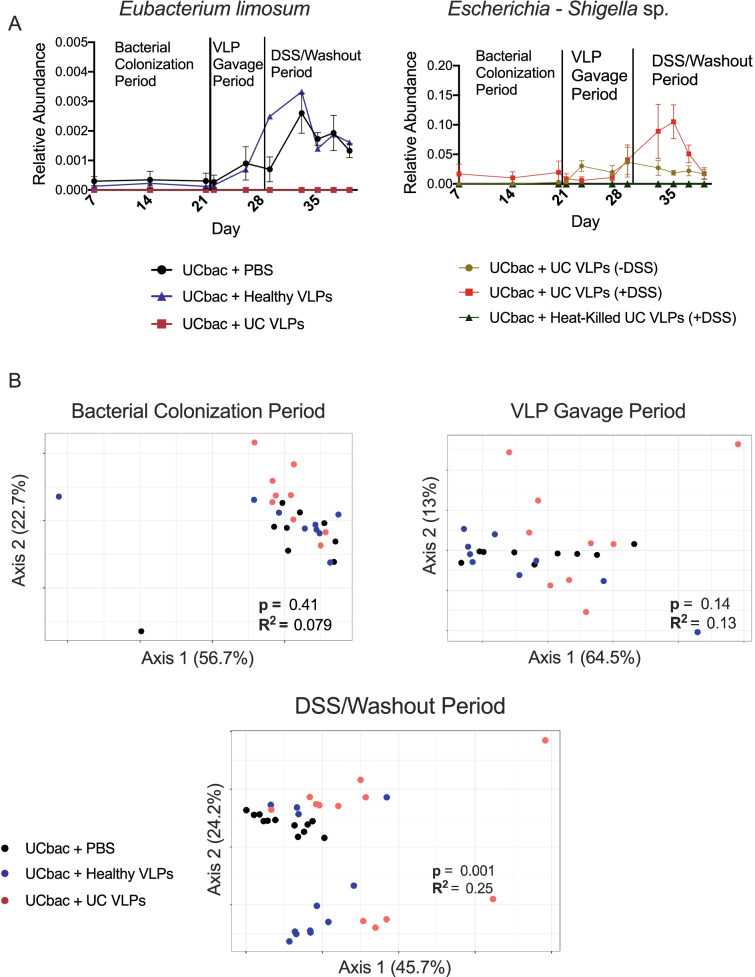
UC VLP administration differentially modulates bacterial community composition in UC-HMA mice. **A** Mean relative abundance of *Eubacterium limosum* (left, data shown is from experiment “B” (trial #1) and *Escherichia-Shigella* sp. (right, data shown is from experiment “C”) in UC-HMA mice over time was determined using 16S rRNA gene sequencing. ANCOM II was used to confirm that these species were differentially abundant between treatment groups [50]. Error bars, SE. **B** Data shown is from experiment ‘B’ (trial #1). PCoA on weighted UniFrac distance between HMA mice during the bacterial colonization period, VLP gavage period, and DSS/washout period. Significant differences between weighted UniFrac distances were assessed using PERMANOVA (*p* ≤ 0.05) (bottom). Samples from all sampling points were included in the PCoA and comparative analyses. Mouse fecal samples in each cage were pooled from 2 mice (*n*=3 cages, 6 mice per treatment group). Dots represent pooled mouse fecal samples at a single sampling point. UCbac, UC-HMA mice

Across the two trials where UC-HMA mice were given multiple doses of healthy VLPs, UC VLPs, or PBS, 2 differentially abundant species (trial #1; *Anaerotruncus* sp., trial #2; *Eubacterium] fissicatena* group sp*.*) were identified in the VLP gavage period before DSS administration, and 12 species (3 species trial #1; 9 species trial #2) were identified in the DSS/washout period (ANCOM cutoff W > 0.6, Supplementary Table S9). Between mice given multiple doses of UC VLPs (+/− DSS) or heat-killed UC VLPs (experiment C), 1 differentially abundant bacterial species was identified in the VLP gavage period (*Blautia hydrogenotrophica*), and 4 differentially abundant species were identified in the DSS/washout period (Supplementary Table S10). Some of these differentially abundant bacterial species have been shown to influence experimental colitis severity or to be differentially abundant in CD or UC patients. For example, *Eubacterium limosum* was significantly reduced in mice given UC VLPs (Fig. 6A, left), consistent with its ability in mouse models to ameliorate DSS colitis severity [60]. In addition, compared to heat-killed UC VLPs, mice given intact UC VLPs showed a significant increase in the proportion of *Escherichia-Shigella* sp. after VLP gavage (Fig. 6A, right). Given that *Enterobacteriaceae* are increased in IBD patients and are thought to exacerbate dysregulated immune responses in IBD [7, 13, 61], these data suggest that UC VLPs may allow for the expansion of this species.

Grouping samples within time periods together, there were no significant differences in bacterial beta-diversity of HMA mice based on the VLP treatment group during the bacterial colonization period and during the following 9-day period where 4 doses of VLPs (healthy, UC, or PBS) were given to these mice (Fig. 6B). In contrast, we observed significant differences in bacterial community composition between treatment groups, and the largest effect size following DSS administration, supporting the idea that these phages have distinct effects on bacterial communities, which are amplified during DSS colitis (Fig. 6B). Similar trends were observed in a second independent trial (Supplementary Fig. S10) and between UC-VLP treated mice and heat-killed controls (Supplementary Fig. S11). In line with these data, performing PERMANOVA analyses on each sampling point in each experiment revealed that significant differences between treatment groups and the largest effect sizes were only observed during the DSS/washout period (Supplementary Tables S11–S13).

In order to investigate whether these greater changes in bacterial diversity following DSS administration were in part due to prophage induction as a result of intestinal inflammation, we also determined the proportion of VLP scaffolds identified as temperate following DSS. Within each treatment group, there was only a modest increase in the relative abundance of VLP scaffolds identified as temperate following DSS (Supplementary Fig. S12). Additionally, to determine whether there was experimental evidence for prophage induction due to direct interactions between DSS and UC bacterial communities, we performed an in vitro prophage induction assay [62–64]. In UC bacterial cultures grown anaerobically in the presence of DSS, we did not detect an increase in VLPs in the bacterial supernatant (Supplementary Fig. S13), suggesting that any DSS-mediated prophage induction occurring in HMA mice is likely not due to direct DSS toxicity to bacterial cells and is likely through DSS-mediated induction of downstream immune responses.

Together, these data indicate that healthy and UC VLPs drive distinct changes in the relative abundance of bacterial species in UC-HMA mice, some of which have been shown to influence experimental colitis severity and IBD disease progression. In addition, while some bacterial species were found to be differentially abundant during the VLP gavage period, differences in bacterial diversity between VLP-treated mice are greatest during DSS colitis, suggesting that intestinal inflammation may provide a more conducive environment for phage-mediated changes in these gut bacterial communities.

### UC VLPs increase colitis severity in HMA mice

**Fig. 7:**
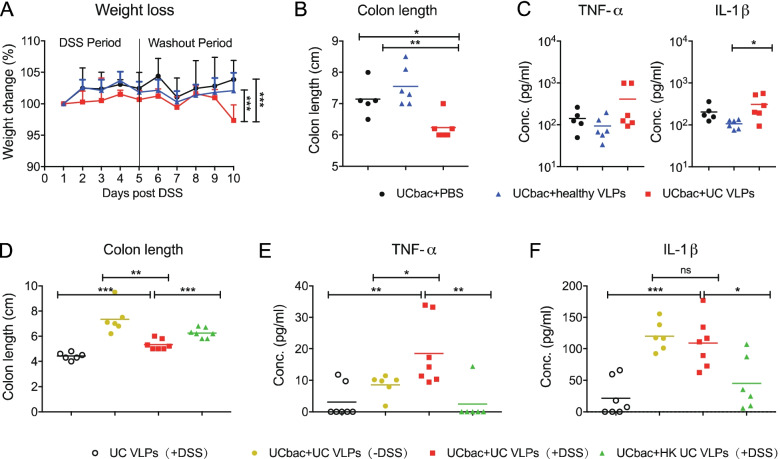
UC VLPs exacerbate the severity of experimental colitis in the presence of gut bacteria. **A**–**C** Data shown is from experiment “B” (trial #1). **D**–**F** Data shown is from experiment “C.” **A** Mean body weight change during experimental colitis induction between the indicated groups. **B** Mean colon length at day 5 post-DSS administration between the three groups. **C** Mean inflammatory cytokine production in colon tissue explant at day 5 post-DSS administration. **D** Mean length of the colon at day 5 post-DSS administration of the following groups: UC bacteria colonized mice treated with UC VLPs with/without DSS challenge, UC bacteria colonized mice treated with heat-killed (HK) UC VLPs and UC VLP treatment alone. **E**, **F** Mean inflammatory cytokine production in colon tissue explants of the indicated groups. **A** Data were analyzed by two-way ANOVA with Bonferroni for multiple comparisons. **B**–**F** Data were analyzed using a two-tailed unpaired parametric *t* test (*p < 0.05, **p < 0.01, ***p < 0.001). Error bars, SD. Data shown from one experiment. Each dot represents an individual mouse. UCbac, UC-HMA mice

Given that the healthy and UC VLPs are distinct in their composition and contribute differently to bacterial community composition, we next tested if they had distinct effects on colitis outcomes. We first determined whether there were differences in colitis severity between UC-HMA mice given multiple doses of healthy VLPs, UC VLPs, or PBS. Upon DSS administration, a slight decrease of body weight could be seen only in mice administered UC VLPs (Fig. 7A). Importantly, at day 10 post-DSS challenge, only the weight of UC-HMA mice given UC VLPs was declining (Fig. 7A). HMA mice given UC VLPs also showed a significant shortening of colon length (Fig. 7B) and an increase of pro-inflammatory cytokine production (Fig. 7C), indicating that UC VLPs can exacerbate the severity of DSS colitis compared to healthy VLPs and the PBS control.

To further investigate the function of UC VLPs on the pathology of DSS-induced colitis, we next assessed DSS colitis severity between UC-HMA mice given (i) UC VLPs with DSS; (ii) UC VLPs without DSS to determine if UC VLPs can cause spontaneous colitis; (iii) heat-killed UC VLPs to determine if intact VLPs are necessary for increased colitis severity; and (iv) GF mice given UC VLPs to determine if increased colitis severity was a result of direct UC VLP-immune interactions (Fig. 7C).

Consistent with our previous results (Fig. 7C), UC-HMA mice given UC VLPs had a significantly shortened colon and produced the most TNF-⍺ and IL-1β from colon explants compared to the other UC-HMA groups (Fig. 7D–F). Notably, the heat-killed UC VLP group mice showed significantly less severe colitis compared to the intact UC VLPs group, suggesting the changes we observe are due to viral infection rather than the presence of phage components alone (Fig. 7D–F). Finally, we found that GF mice given UC VLPs prior to DSS treatment exhibited the greatest colon shortening, yet least inflammatory cytokine production (Fig. 7D–F). These results are similar to our observations of increased colonic shortening in GF mice compared to HMA mice (Supplementary Fig. S14) and suggest that, without bacterial hosts, phages cannot modulate colitis outcomes.

## Discussion

Phages are known regulators of bacterial diversity and metabolism in several environments, including the mammalian gut [18, 65, 66]. In IBD, metagenomic analyses have revealed that the virome composition of CD and UC patients is altered in comparison to non-IBD controls [37–40]. However, experimental work investigating the interactions between phages, intestinal bacterial communities, and immune responses in the context of IBD is limited [67]. Given the importance of gut bacterial communities in human health, phage-mediated changes of the microbiota are thought to have important physiological consequences [26]. Here, we show that VLPs isolated from healthy volunteers and UC patients differentially modulate the composition of the gut microbiota, and that administration of multiple doses of UC VLPs increases colitis severity in UC-HMA mice.

Four doses of UC VLPs and healthy VLPs significantly altered the fecal bacteriome of UC-HMA mice compared to PBS and heat-killed UC VLP-treated controls. This was not seen when a single dose of UC VLPs was used, supporting the idea that the regulatory effect of phages in the mammalian gut is dose-dependent [23]. The differences in the effects of multiple doses compared to single doses of VLPs on bacterial community composition and downstream disease outcomes have important implications for targeted or whole community phage therapy approaches in the gut [26].

Interestingly, we only observed significant differences in bacterial beta-diversity during the DSS/washout period, highlighting that any initial VLP-mediated effects are amplified during the DSS colitis/washout period. The disrupted colonic mucus layer, potential changes in bacterial growth rate, and/or innate immune cell-derived mediators in the inflamed gut could all provide a more conducive environment for increased phage-host interactions [56]. Given these drastic changes in the gut environment during inflammation, it is possible that this facilitates a switch from Piggyback-the-Winner dynamics, common in the mammalian gut, to Kill-the-Winner dynamics [56]. Changes in microbial expression profiles in IBD patients [68] and in murine experimental colitis [69] could also alter bacterial susceptibility to phage infection.

The use of DSS colitis as a model of inflammation is limited in that the immune responses elicited and the exaggerated damage to the epithelium differ from clinical UC [70]. However, we induced a mild colitis, as HMA mice given 2% DSS experienced minimal weight loss and blood in their stool. In addition, the reproducibility and temporal control offered by DSS were valuable in our approach to study the effects of VLPs on bacterial communities in the context of broad inflammatory progression and changes. Thus, our data provide the framework for future studies investigating phage-host interactions during inflammation. Given the modest increase in the relative abundance of temperate phages observed across treatment groups following DSS, prophage induction resulting from DSS-induced inflammation could also contribute to these differences in bacterial diversity. Further to this point, we are likely underestimating the number of phages classified as temperate. This limitation is, in part, because some samples collected during DSS exposure could not be used for VLP shotgun sequencing due to DSS-mediated inhibition of DNA polymerase required for multiple displacement amplification (MDA). Interestingly, while we did observe differences in bacterial community diversity based on VLP treatment, there were only limited differences in bacterial activity or damage in VLP treated mice, suggesting that these changes were too modest to detect at a community level.

As previously observed, there were differences in virome composition between the healthy and UC fecal VLP and bacterial inoculum [7, 37–40]. However, contrasting with previous metagenomic analyses of IBD viromes, there was not a high relative abundance of dsDNA *Caudovirales* phages in our pooled UC VLP inoculum. Instead, the UC virome was dominated by *Microviridae*. Our limited sample size to prepare the pooled phage inoculums, the high inter-individual differences in gut viromes [30], differences in virome composition based on viral enrichment and DNA extraction strategies [28, 71, 72], and the bias in the amplification of ssDNA viruses using multiple-displacement amplification (MDA) [73, 74] are all possible causes that could explain these differences between the virome composition in our dataset and previously published viromes of IBD patients. Still, our observations of increased richness and proportions of temperate phages in the UC VLP inoculum, and alterations in Bacteroidetes, Actinobacteria, and Proteobacteria in the UC bacterial inoculum are consistent with previously reported UC-specific alterations of the microbiota [37, 46–48]. While our strategy to pool the healthy and UC bacterial and VLP inoculums comes with the loss of inter-individual genomic information between samples within a given treatment group, pooling inoculums maintains intra-specific information while minimizing variation during transplantation, especially important given the stochasticity during microbiota acquisition [75] and low percentage of retained taxa following transplantation [76]. Importantly, we assessed the composition of the pooled viral and bacterial inoculums in addition to following changes in the microbiota of HMA mice over time, thus allowing us to assess the efficacy of bacterial and VLP engraftment [77]. Additionally, despite the limitations of this approach, compared to previous studies that have investigated VLP-mediated changes to bacterial diversity or disease severity in mice [21, 23, 24], our use of HMA mice and human-derived VLPs adds translatability of our findings to the microbiota alterations observed in UC patients.

Importantly, we observed differences in viral beta-diversity post-VLP gavage and identified scaffolds transferred from the pooled VLP inoculums to HMA mice, suggesting that phages within the healthy and UC inoculums were able to successfully engraft in recipient mice. While VLP shotgun sequencing could not be performed during DSS colitis, differences in viral diversity persisted into the DSS/washout period, suggesting that viromes of mice given healthy and UC VLPs were divergent before and after inflammation. In addition, the post-gavage viromes of healthy and UC VLP-treated were characterized by distinct phage-host interactions, as predicted by CRISPR spacer matches. The treatment-specific differences in the predicted bacterial hosts that these phages target are in support of our observations that healthy and UC VLPs have divergent effects on gut bacterial communities. The particular observation that healthy VLPs successfully engrafted in HMA mice colonized with UC bacterial communities is consistent with previous observations of phage “cross-reactivity” between different individuals or disease states and likely supports the idea that some phages in the gut have host ranges at the species rather than the strain level [21, 23, 24, 78, 79]. Notably, our functional annotation of viral scaffolds revealed no obvious differences in the auxiliary metabolic genes present between treatment groups (data not shown), suggesting shared potential functionality between viromes.

Regardless of whether HMA mice were given a single dose of healthy or UC VLPs, mice colonized with fecal bacteria isolated from UC patients had increased colitis severity compared to mice colonized with fecal bacteria from healthy donors. This finding is consistent with previous reports showing exacerbation of DSS-induced and T cell-mediated colitis following administration of gut microbiota from IBD patients to germ-free mice [51, 52]. While these studies associated an increase of IL-17 producing CD4+ T cells to disease severity, anti-viral immunity is more commonly associated with type 1 immunity and interferon production. As such, an impact of the virome on T cell immunity is likely indirect via changes to bacteria-derived products that promote Th17 cell differentiation. Nevertheless, phages express pathogen-associated molecular patterns that, if accessed by host immune cells, may directly alter tissue inflammation [80]. Although we administered UC VLPs to GF mice in order to investigate this scenario, VLPs were cleared in the absence of bacterial hosts, resulting in undetectable changes to the host immune response.

Two important limitations in using HMA mice to monitor disease outcomes are that (1) only a fraction of human fecal bacteria can colonize GF mice [76] and (2) there is a reported colonization bias towards Bacteroidetes [76]. Despite these limitations, we identified differences in the relative abundances of certain bacterial species in mice after colonization with healthy or UC fecal bacteria, which could explain the differences in colitis severity between these two groups. This included *Akkermansia* sp*.*, which are reduced in IBD patients and have been shown to be protective in the context of DSS colitis [12], and *Escherichia-Shigella* sp*.,* which are thought to exacerbate the pro-inflammatory immune responses in IBD [81]. We speculate that the increased colitis severity observed in UC-HMA mice given UC VLPs compared to mice given PBS, heat-killed UC VLPs, or healthy VLPs may be due to alterations of the gut bacterial communities resulting from phage predation. Some of the bacterial species reduced in relative abundance by UC VLPs, such as *E. limosum* [60], are thought to be producers of short-chain fatty acids (SCFAs), which are important in promoting epithelial barrier function, antimicrobial peptide production, and induction of immunomodulatory Tregs [3, 9]. Given that IBD patients have reduced fecal SCFA concentrations [82, 83], these data support the idea that UC VLPs may cause increased colitis severity by depleting these SCFA-producing bacteria. In support of this idea, we found that the reduction of *E. limosum* during the DSS/washout period in UC VLP-treated mice coincided with an enrichment of phages predicted to infect *Eubacterium*. However, given the current limitations in bioinformatically matching phages to their hosts (we were only able to assign predicted hosts to ~ 20–30% of our viral scaffolds) and the diverse interbacterial interactions in the gut, it is difficult to definitively attribute the phage predation of specific bacterial taxa to an altered disease pathology. Notably, UC VLP administration also led to an increase in the relative abundance of *Escherichia*-*Shigella* sp*.*, possibly an indirect result of phage predation and its subsequent modulation of the gut microbiota through interbacterial interactions, as recently described by Hsu et al. [19]. Consistent with the notion that Proteobacteria can contribute to IBD pathogenesis [13], our data indicate that phage regulation of bacterial communities promotes the expansion of immune-activating, potentially pathogenic bacteria. In agreement with our earlier observations, Khan Mirzaei et al. [79] showed that phages from stunted children promoted the in vitro expansion of Proteobacteria, which are important in contributing to the nutritional deficiencies of this disease. The observation that VLPs isolated from the patients of two distinct diseases allowed for the expansion of Proteobacteria across different ages and experimental settings has important relevance for virome alterations in inflammatory diseases and warrants further investigation. While it is possible that endotoxin remaining in the VLP preps also contributed to the observed differences in colitis severity, mice given heat-killed UC VLPs displayed reduced colitis severity compared to intact UC VLPs, suggesting that LPS contamination does not explain these effects.

## Conclusions

While several studies have outlined gut virome alterations in IBD patients, our observations that fecal VLPs from UC patients exacerbate DSS colitis severity suggest that these alterations could be important for IBD pathogenesis and gut inflammation. This action may be either through phage-mediated changes in the microbiota or by direct interactions with the intestinal immune system. Overall, the power of experimental in vivo cross infections performed here allowed us to highlight a causal role for phages in modulating gut bacterial communities and disease outcome.

## Methods

### Lead contact

Further information and requests for resources and reagents should be directed to and will be fulfilled by the lead contact, Corinne Maurice (corinne.maurice@mcgill.ca).

### Subject details and sample collection

For all experiments, fecal samples were collected from 3 UC patients (mean age: 38.66 ± 12.97 SD, mean BMI: 28.89 ± 8.14 SD) in remission and 3 unrelated healthy controls (mean age: 42.33 ± 13.65 SD, mean BMI: 23.40 ± 3.91 SD). Samples were stored at − 80 °C until processed. Participants of this study were only included if they were older than 18 years and had not taken antibiotics in the 3 months prior to sample collection. UC patients were excluded if they were administered treatments other than immunosuppressants. Human studies were performed with approval of the McGill Ethics Research Board (REB #A04-M27-15B).

### Mice

Six- to 12-week-old female and male germ-free (GF) C57BL/6 mice were purchased from Charles River Laboratories (Wilmington, North Carolina) and maintained in flexible film isolators at McGill University. All mice had unlimited access to autoclaved mouse breeder’s diet and water. All mouse experiments were carried out in accordance with the approved McGill University animal use protocol (7977).

### Experimental model

HMA mice were used to assess how VLP administration impacts the gut bacterial communities and colitis severity. A schematic of each experiment performed, and associated timelines are summarized in Fig. 1. Bacterial communities derived from the feces of UC patients or healthy controls were administered to GF mice by oral gavage (200 μL, 1–3 × 10^8^ cells/mL). All colonized HMA mice were maintained on the same sterilized diet and water as before colonization. During the 3-week bacterial reconstitution period, random fecal samples were weighed and collected from each individual cage every week to monitor the reconstitution. Stool DNA was extracted by using the QIAGEN QIAamp DNA stool mini Kit under a sterile biosafety cabinet. Real-time PCR amplification of the V4 region of the 16S rRNA gene was performed and the concentration of purified PCR products was quantified by NanoDrop spectrophotometer as a standard template. (forward primer: aggattagataccctggta and reverse primer: rrcacgagctgacgac). The load of bacterial DNA in feces was estimated by 16S rRNA gene qPCR, and the concentration was calculated according to the dilution of series of template, and normalized to the stool weight. Following a period of 3 weeks of bacterial reconstitution, 1 or 4 doses of VLPs derived from the feces of UC patients or healthy controls were administered to the HMA mice by oral gavage at equivalent concentrations to the bacterial dose given [84] over a 9–10 day period. Controls consisted of the administration of PBS or heat-killed VLPs to HMA mice following bacterial reconstitution. Following VLP treatment, 2% DSS was administered to the mice for 5 days, followed by a 5-day washout period. All mice were sacrificed at the end of the 5-day DSS/washout period, and mouse colons were removed to determine DSS colitis severity. Throughout the course of the experiments, mouse fecal pellets were collected to determine bacterial and VLP abundance, bacterial activity, and damage; to monitor disease activity index; and to determine bacterial VLP community composition.

### Processing of human fecal samples

VLPs and bacterial communities were extracted and processed from human fecal samples, as described by Khan Mirzaei et al. [79] with some modifications. To acquire community-level information and obtain enough material for multiple mouse experiments, two grams of each frozen healthy or UC fecal sample were pooled separately, thawed under anaerobic conditions, resuspended with reduced PBS (rPBS), containing 1 μg ml^−1^ resazurin sodium salt and 1 mg ml^−1^ L-Cysteine; final concentrations, thoroughly vortexed, and centrifuged at 800*g* for 1 min at 22°C to remove large debris. Pooling samples still allows for intraspecific genomic information of bacterial and viral communities, despite a loss of inter-individual differences [85]. The resulting supernatant was centrifuged at 7000*g* at 22 °C for 1 h to separate bacterial and VLP communities. Following resuspension in rPBS, bacterial concentration was determined using flow cytometry (see below) [57]. The VLP-containing supernatant was filtered through a 0.2-μm sterile syringe filter (Millex-GP, Millipore Sigma, USA) concentrated by centrifugation (35,000*g*, 4°C) for 3 hr and resuspended in SM buffer. VLP concentration was determined using epifluorescence microscopy (see below) [22]. DNA extractions were performed on aliquots of each pooled VLP and bacterial preparation for downstream sequencing analysis (see below). As a control, one aliquot of the extracted VLPs was heat-inactivated by incubating the solution at 95 °C for 20 min, followed by DNAse (Ambion DNAse I, Thermo-Fisher Scientific, USA) treatment for 2 h [22]. Bacterial and viral abundances were confirmed prior to each gavage, and two hundred microliters of bacterial and VLP communities were administered to mice by oral gavage at equal concentrations (1–3 × 10^8^ VLPs or bacterial cells/mL).

### Induction of DSS colitis and disease activity index (DAI) evaluation

HMA and GF mice received 2% (w/v) DSS (MP Biomedicals) in drinking water for 5 days prior to regular drinking water for another 5 days. The amount of DSS intake per mouse was recorded. Each individual mouse was weighed to determine percentage weight changes. Fecal samples were taken from each cage to clinically monitor for rectal bleeding and diarrhea. Hemoccult SENSA kit (Beckman Coulter) was used to assess rectal bleeding as per the manufacturer’s instructions. All parameters were evaluated every 3 days.

### Histological evaluation of colitis

HMA mice were anesthetized and sacrificed five days after completing DSS administration. 0.5 cm of the distal colon was excised, rinsed with saline solution, fixed in 10% formalin, and embedded in paraffin. Sections of 4 μm were stained with H&E by the Goodman Cancer Research Centre Histology Facility and assessed for histological changes in a blinded manner.

### Pro-inflammatory cytokine ELISA

The top 0.5 cm of the colon was harvested and cut longitudinally, washed in PBS and excess liquid was removed. The tissue was then weighed, placed in 400 μL of culture medium RPMI1640 supplemented with 10% *fetal bovine serum,* and cultured in 37 °C incubator overnight. The supernatant was collected and transferred into a 1.5 mL tube and centrifuged for 5 min at 13,000 rpm for 4 °C. The supernatant was collected, stored at −20 °C, and used for ELISA. The mouse IL-6, IL-1β, and TNF-α ELISA Kits from Invitrogen (ThermoFisher) were used as per the manufacturer’s instructions.

### Colon cell extraction

The middle 5 cm of the colon was used to generate single-cell suspensions of lamina propria cells. The tissue was cut longitudinally and washed in cold Hank’s Balanced Salt Solution (HBSS) + EDTA buffer. The tissue was then cut into 0.5–1-cm sections, placed in HBSS + EDTA buffer, and incubated at 37 °C for two 20-min periods with washing between incubations. The tissue was then washed twice with cold HBSS buffer and digested in 5mL of digestion buffer (RPMI 1640 supplemented with 10% FBS, 200 U/mL of Collagenase VIII) for 25 min at 37 °C. After digestion, the tissue was passed through a 100-μm filter and resuspended in PBS + 5% FBS for counting using Trypan blue and flow cytometry analysis.

### Flow cytometry of colon cells

Colon cell suspensions were incubated with a fixable Viability dye (eFluor 506, eBioscience) for 25 min at 4 °C. Cells were then incubated with Fc block (7 min at 4 °C), followed by staining (for 30 min at 4 °C) with the following antibodies in appropriate combinations of fluorophores. From Invitrogen: CD45.2 (104). From Biolegend: CD11b (M1/70), Ly6c (HK1.4), Ly6g (1A8). Data were acquired with a FACS Canto II or LSR Fortessa (BD Biosciences) and analyzed using FlowJo software (TreeStar).

### Processing mouse fecal samples for microbiota analyses

Mouse fecal samples were processed similar to human fecal samples, with some modifications.

On each sampling date, fecal pellets were collected from each mouse and samples belonging to the same cage were pooled in order to maximize the amount of biological material obtained. Pooled pellets were resuspended in rPBS under anaerobic conditions, thoroughly vortexed, and centrifuged at 800*g* for 1 min at 22°C to remove large debris. The resulting supernatant was centrifuged at 6000*g* for 5 min at 22°C to separate VLP and bacterial communities. VLP-containing supernatant was filtered through a 0.2-μm sterile syringe filter (Millex-GP, Millipore Sigma, USA) to remove the remaining bacteria, and phage abundance was determined using epifluorescence microscopy (see below). The bacterial pellet was washed twice and resuspended in rPBS under anaerobic conditions. The concentration and proportion of active and damaged bacterial cells were determined using flow cytometry (see below). For the remaining fecal pellets from each cage, bacterial and VLP communities were separated, as described above. Following separation, bacterial and VLP DNA were extracted separately.

### Bacterial DNA extraction and 16S rRNA gene sequencing analyses

Bacterial DNA was extracted from human and mouse feces-derived bacterial supernatant using the DNeasy Powersoil Kit (Qiagen, USA) according to the manufacturer’s instructions. Data included from healthy and UC bacterial inoculums are from four and five technical sequencing replicates, respectively. The V4 region of the 16S rRNA gene was amplified using the 515F/806R primers, and pooled amplicons were sequenced on an Illumina Miseq with 250 bp paired-end technology at the Genome Québec *Centre d’expertise et de services* core facility [86]. 16S rRNA gene sequencing analysis was performed using the QIIME2 platform (v. 2021.11) and the phyloseq package (v.1.3.4) [87, 88]. Read-trimming, removal of chimeric reads, merging of paired-end reads, and inference of amplicon sequence variants were performed using DADA2 [89]. In each sequencing run, read depth was rarefied to the lowest number of reads that a sample contained in that run. Bacterial diversity between treatment groups was assessed using Weighted UniFrac and PCoA. Taxonomic identification was performed using the QIIME2 feature classifier, training a Naives-Bayes Classifier on the Silva 138 database [90]. The statistical framework, ANCOM II, was used to identify differentially abundant bacterial taxa between treatment groups during different time periods [50]. Mouse cage was added as a random effect in the linear model design for ANCOM II to account for repeated sampling. In order to disentangle the effects of VLP treatment and possible isolator-specific colonization biases, bacterial species that were found to be differentially abundant during the bacterial colonization period were removed from Supplementary Tables S9 and S10. In addition, each differentially abundant bacterial species was assigned a ranking (1–3) based on likelihood that differences in relative abundance were due to VLP treatment or isolator effect (1: likely due to phage treatment, 2: possibly due to phage treatment, 3: likely due to isolator effect). Species classified as “likely due to phage treatment (1)” included species, where reads were detected in a majority of cages in all treatment groups. Species classified as “possibly due to VLP treatment” (2) included species that were found to be differentially abundant in the VLP gavage and/or DSS/washout period and where reads were not detected in a majority of cages in all treatment groups during the bacterial colonization period. Any differentially abundant species that did not meet the above criteria were classified as “likely due to isolator effect” (3).

### VLP DNA extraction, amplification, and sequencing

VLP enrichment and subsequent DNA extraction was performed on VLP inoculum samples and HMA mouse fecal samples (only from experiment B (trial #1)) as described by Reyes et al. [22] with some modifications. Briefly, feces-derived VLP supernatant (prepared as described above) was incubated with lysozyme (45 min at 37 °C, 50 mg·mL^−1^), Ambion™ Dnase I (1 h at 37 °C, 2U), and proteinase K (1 h at 37 °C, 20 mg·mL^−1^). After addition of 5M NaCl and 10% cetyltrimethylammonium bromide (CTAB)/0.7M NaCl solutions, samples were transferred to phase lock gel tubes (QuantaBio, USA) with an equal amount of phenol:chloroform:isoamyl alcohol (25:24:1 v/v, pH = 8.0, ThermoFisher Scientific, USA). The upper aqueous, DNA-containing, the layer was transferred to a new tube and left to precipitate overnight at − 80 °C in 100% ice-cold ethanol. Precipitated samples were then purified with the Zymo DNA Clean & Concentrator 25 kit (Zymo Research, USA). Following purification, 1uL of DNA was amplified in triplicate using MDA, using the GenomiPhi V3 DNA amplification kit (Cytiva, USA). Amplified products were pooled, and DNA concentrations were quantified with the Qubit dsDNA high-sensitivity (HS) assay kit (ThermoFisher Scientific, USA). In total, 27/90 samples from HMA mice in experiment “B” (trial #1) were excluded from sequencing due to insufficient DNA yield, including all 18 samples (2 sample collection dates) during the DSS colitis period. For virome analyses of pooled human inoculums, 4 (healthy) and 5 (UC) technical replicates were sequenced from the same pooled fecal samples. Paired-end Illumina shotgun sequencing libraries were prepared from purified VLP-derived DNA at the Genome Québec *Centre d’expertise et de services* core facility. Barcoded libraries which passed quality control were pooled and sequenced on an Illumina MiSeq with 250 bp paired-end sequencing technology.

### VLP abundances

VLPs were enumerated using epifluorescence microscopy [33]. Briefly, human or mouse-derived VLP supernatant was diluted in TE buffer and fixed with 1% formaldehyde for 15 min. Fixed VLPs were filtered onto 0.02-μm filter membranes in triplicate (Anodisc, GE Healthcare, USA) and were stained with SYBR-Gold (2.5X concentration). On average, 25 fields of view of the stained filters were visualized and counted in triplicate using an epifluorescence microscope (Zeiss, Axioskop).

### Bacterial abundance and physiology

The concentration of bacterial cells and the proportion of active and damaged bacterial cells in human and mouse feces was determined using flow cytometry, as described previously [57]. Briefly, feces-derived bacterial supernatant was diluted in rPBS anaerobically, stained with SYBRGreen I (1x concentration, 15 min) or PI (1x concentration, 10 min), and counted using flow cytometry on a FACSCanto II (BD, USA) equipped with a 488-nm laser (20 mW) and 530/30 and 585/42 detection filters. Five-to-ten microliters of green fluorescent reference beads of 3.0–3.4 μm (Rainbow beads, BD Biosciences) were added to each flow cytometry tube prior to acquisition to calculate bacterial concentration. The concentration of the Rainbow beads was determined by calibration with Trucount tubes (BD, USA) for each sampling date [91]. Total bacterial abundance was determined by SYBRGreen I staining. As previously described [91], relative nucleic acid content was used as a proxy for bacterial activity: active cells, containing more nucleic acid, were identified by their higher levels of green fluorescence. Bacterial membrane damage was assessed by PI staining: cells with high red fluorescence have lost their membrane integrity. All stainings were done in triplicates. Gating was performed using the FlowJo analysis software (v10.6.1). The gating strategy for bacterial metabolic activity is shown in Supplementary Fig. S5A, and the gating strategy for membrane damage is shown in Supplementary Fig. S6A.

### Prophage induction assay

In order to determine whether DSS could directly cause prophage induction of fecal bacteria, we performed an in vitro induction assay [62]. Briefly, pooled UC fecal bacteria were grown anaerobically in triplicate at 37 °C, mixing every 15 min, in reduced Brain Heart Infusion broth (BBL BD, Mississauga, ON, Canada) supplemented with hemin (5 μg/mL) and vitamin K (1 μg/mL). At the early exponential phase, DSS was added to the growth media at final concentrations of 0% (H_2_O added), 1%, 2%, or 5%. Bacterial growth was monitored every 15 min for 14 hr, measuring OD_600_ (Epoch 2 microplate spectrophotometer, Biotek Instruments, Winooski, VT, USA) until stationary phase was reached. Bacteria were pelleted (2000×*g*, 15 min, 22 °C), and VLP-containing supernatant was fixed with 1% formaldehyde and enumerated using epifluorescence microscopy. Induction was identified through a concomitant significant decrease in bacterial abundance and an increase in VLPs.

### Bioinformatic analysis of VLP sequence data

Virome analysis was performed on shotgun-sequenced VLP DNA, as described in Supplementary Fig. S1. Briefly, raw reads (mean reads mouse samples: 176,955 ± 21,451 SD; mean reads human VLP inoculums: 1,176,926 ± 79,038 SD) were first trimmed using Trimmomatic (v0.38) [92]. De novo assembly of sequencing reads was carried out with metaSPAdes (v3.12.0) [93], using default settings. Scaffolds from each sample’s assembly were then pooled and those less than 3 kb in length were removed [30]. Scaffolds were then classified as viral if they met any of the following criteria: (1) categorized as viral by VirSorter v1.0.6 (virome decontamination setting, viromes + Gut Virome Database v1.7.2018, keeping only category 1 and 2 hits) [28]; (2) categorized as viral by VIBRANT v1.2.1 [29] with the virome mode enabled; (3) BLASTn alignment to the Viral RefSeq database v. 207 (*e*-value ≤ 10^−10^, covering ≥ 90% of the scaffold’s length, and over 50% nucleotide identity). Scaffolds that were classified as viral were retained for downstream taxonomic and diversity analysis. Scaffolds classified as viral from mouse samples and scaffolds classified as viral from the pooled human inoculum samples were pooled, and scaffolds with 90% nucleotide identity over 70% of the shorter scaffold’s length were removed using CD-HIT-EST v4.8.1 [94] to form the non-redundant scaffolds library. vConTACT2 v0.9.10 was used to form viral clusters from viral scaffolds [44]. These clusters were added to the scaffold metadata used for downstream analysis. Demovir (https://github.com/feargalr/Demovir) was used to assign taxonomic identities to the viral scaffolds. This tool uses a voting approach for taxonomy based on protein homology to a viral subset of the TrEMBL database from UniProt. Additionally, scaffolds were identified as being a crAss-like phage if they had BLASTn alignments to a crAssphage database with an *e*-value ≤ 10^−10^, covering ≥ 90% of the scaffold’s length, and over 50% nucleotide identity [30, 95]. We used CrisprOpenDB, which uses BLASTn alignment to a CRISPR spacer database to assign bacterial genus predictions for each viral scaffold [49]. Default settings were used, allowing up to 2 mismatches between a scaffold and spacer. Using BACPHLIP, scaffolds were identified as temperate or virulent [45]. We used Bowtie2 v2.3.5 [96] to map trimmed reads back to assembled viral scaffolds. SAMtools v1.9 [97] was used to convert SAM to BAM files. Custom Python scripts were used to create scaffold metadata files and abundance matrices. These scripts are available at the GitHub (https://github.com/MauriceLab/phage_colitis). Scaffolds were only included in analyses for a given sample if coverage was ≥ 1× over ≥75% of the scaffold length [98]. In order to determine differentially abundant viral scaffolds, raw counts matrices were input into DESeq2 v.1.3.0 [59]. The standard DESeq2 workflow was performed to analyze treatment-specific differences before and after VLP gavage, while controlling for repeated sampling of mouse cages. Poscounts were used for estimation of size factors and scaffolds with adjusted *p* values < 0.01 and with log_2_fold changes greater or less than 1 were considered differentially abundant [99]. Bray-Curtis dissimilarity and Jaccard distances for diversity analyses were performed on matrices normalized by total coverage using Vegan v2.5-7. Visualization of NMDS and relative abundance plots was performed using the ggplot2 v3.3.3 package.

### Quantification and statistical analysis

Data were analyzed using GraphPad Prism software (v 8.4.3). Specific tests for determining statistical significance are indicated in the figure legends. *P* values < 0.05 were considered statistically significant, except for DESeq2 virome analyses, where *p* values < 0.01 and values with log_2_fold change greater or less than 1 were considered statistically significant. Differences in viral diversity were assessed using NMDS of Bray-Curtis dissimilarity and Jaccard distance in R v.4.0.5 (R Core Team, 2021) using the Vegan v2.5-7 and ggplot2 v3.3.3 packages. Adonis PERMANOVA was used to determine significant differences in Bray-Curtis Dissimilarity between the viromes of HMA mice given healthy VLPs, UC VLPs, or PBS, using the Vegan v2.5-7 package. Differentially abundant bacterial species were identified using ANCOM II. PCoA on weighted UniFrac distances were used to determine differences in bacterial diversity between HMA mice. Significant differences on weighted UniFrac distance between HMA mice was determined using adonis PERMANOVA using phyloseq v.1.3.4 [87].

## Supplementary Information

**Additional file 1: Supplementary Figure S1.** Virome analyses on inoculum and mouse VLPs. Related to Figs. 2 and 5. **Supplementary Figure S2.** Experimental colitis severity of UC-HMA and healthy-HMA mice given a single dose of healthy or UC VLPs. Related to Fig. 3. **Supplementary Figure S3.** Viral and bacterial abundance in UC-HMA mice given UC or healthy VLPs. Related to Fig. 4. **Supplementary Figure S4.** Viral and bacterial abundance in UC-HMA mice given intact or heat-killed UC VLPs. Related to Fig. 4. **Supplementary Figure S5.** Bacterial activity in HMA mice given UC or healthy VLPs. Related to Fig. 4. **Supplementary Figure S6.** Bacterial damage in HMA mice given UC or healthy VLPs. Related to Fig. 4. **Supplementary Figure S7.** Richness of viral scaffolds and viral clusters in UC-HMA mice given healthy or UC VLPs. Related to Fig. 5. **Supplementary Figure S8.** Jaccard distance to pooled healthy and UC VLP inoculums over time in UC-HMA mice given UC or healthy VLPs. Related to Fig. 5. **Supplementary Figure S9.** NMDS on Bray-Curtis dissimilarity of viral clusters between UC-HMA mice given UC or healthy VLPs. Related to Fig. 5. **Supplementary Figure S10.** PCoA on weighted UniFrac distance between UC-HMA mice given UC or healthy VLPs. Related to Fig. 6. **Supplementary Figure S11.** PCoA on weighted UniFrac distance between UC-HMA mice given UC VLPs or heat-killed UC VLPs. Related to Fig. 6. **Supplementary Figure S12.** Relative abundance of temperate phages. Related to Fig. 6. **Supplementary Figure S13.** DSS does not induce UC gut bacterial prophages in vitro. Related to Fig. 6. **Supplementary Figure S14.** Human microbiota protects mice from experimental colitis. Related to Fig. 7. **Supplementary Table S2.** PERMANOVA and effect size of weighted UniFrac distances of bacterial communities between UC-HMA mice given a single dose of healthy VLPs or UC VLPs. Related to Fig. 3. **Supplementary Table S3.** PERMANOVA and effect size of weighted UniFrac distances of bacterial communities between healthy-HMA mice given a single dose of healthy VLPs or UC VLPs at each sampling point. Related to Fig. 3. **Supplementary Table S4.** PERMANOVA and effect size of weighted UniFrac distances of bacterial communities between UC-HMA mice and healthy-HMA mice at each sampling point. Related to Fig. 3. **Supplementary Table S7.** PERMANOVA and effect size of Bray-Curtis dissimilarity on viral scaffolds and viral clusters between UC-HMA mice given healthy and UC VLPs. Related to Fig. 5. **Supplementary Table S9.** Differentially abundant bacterial species during in HMA mice given healthy VLPs, UC VLPs, or PBS. Related to Fig. 6. **Supplementary Table S10.** Differentially abundant bacterial species in HMA mice given UC VLPs (+/- DSS), or heat-killed UC VLPs. Related to Fig. 6. **Supplementary Table S11.** PERMANOVA and effect size of weighted UniFrac distance on bacterial communities between UC-HMA mice given healthy VLPs, UC VLPs or PBS at each sampling point. Related to Fig. 5. **Supplementary Table S12.** PERMANOVA and effect size of weighted UniFrac distance on bacterial communities between UC-HMA mice given healthy VLPs, UC VLPs or PBS at each sampling point. Related to Fig. 5. **Supplementary Table S13.** PERMANOVA and effect size of weighted UniFrac distance on bacterial communities between UC-HMA mice given UC VLPs (+DSS), UC VLPs (-DSS) or heat-killed UC VLPs (+DSS) at each sampling point. Related to Fig. 5.

**Additional file 2: Supplementary Table S1.** VLP scaffolds and bacterial phyla and genera in pooled healthy and UC inoculums. Healthy VLP and UC VLP inoculum scaffolds and annotations (tab 1), bacterial phyla relative abundance (tab 2), bacterial genera relative abundance (tab 3). Related to Fig. 2.

**Additional file 3: Supplementary Table S5.** Differentially abundant bacterial species between healthy-HMA and UC-HMA mice. Related to Fig. 3.

**Additional file 4: Supplementary Table S6.** VLP scaffolds shared between the VLP inoculums and HMA-mice post VLP gavage. Scaffolds shared between the healthy VLP inoculum samples and mice given healthy VLPs (tab 1). Scaffolds shared between the UC VLP inoculum samples and mice given UC VLPs (tab 2). Related to Fig. 5.

**Additional file 5: Supplementary Table S8.** Differentially abundant viral scaffolds between VLP treatment groups. UC VLPs vs. PBS control, phage gavage period (tab 1). Healthy VLPs vs. PBS control (tab 2). Related to Fig. 5.

## Data Availability

Custom scripts and code used for data analysis are available at https://github.com/MauriceLab/phage_colitis. Sequencing reads for bacterial 16S rRNA gene sequencing and VLP shotgun metagenomics are available on NCBI SRA. Bacterial 16S rRNA gene sequencing reads are available using accession number: PRJNA734661. VLP shotgun metagenomic reads are available using accession number: PRJNA732769. This study did not generate new unique reagents.
