# Supplementary material for: Transplantation of bacteriophages from ulcerative colitis patients shifts the gut bacteriome and exacerbates the severity of DSS colitis
Source: Microbiome. 2022 Jul 8;10:105. doi: 10.1186/s40168-022-01275-2 (PMC9264660; doi:10.1186/s40168-022-01275-2)
Supplement: Supplementary file 2 — Additional file 1: Supplementary Figure S1. Virome analyses on inoculum and mouse VLPs. Related to Figs. 2 and 5. Supplementary Figure S2. Experimental colitis severity of UC-HMA and healthy-HMA mice given a single dose of healthy or UC VLPs. Related to Fig. 3. Supplementary Figure S3. Viral and bacterial abundance in UC-HMA mice given UC or healthy VLPs. Related to Fig. 4. Supplementary Figure S4. Viral and bacterial abundance in UC-HMA mice given intact or heat-killed UC VLPs. Related to Fig. 4. Supplementary Figure S5. Bacterial activity in HMA mice given UC or healthy VLPs. Related to Fig. 4. Supplementary Figure S6. Bacterial damage in HMA mice given UC or healthy VLPs. Related to Fig. 4. Supplementary Figure S7. Richness of viral scaffolds and viral clusters in UC-HMA mice given healthy or UC VLPs. Related to Fig. 5. Supplementary Figure S8. Jaccard distance to pooled healthy and UC VLP inoculums over time in UC-HMA mice given UC or healthy VLPs. Related to Fig. 5. Supplementary Figure S9. NMDS on Bray-Curtis dissimilarity of viral clusters between UC-HMA mice given UC or healthy VLPs. Related to Fig. 5. Supplementary Figure S10. PCoA on weighted UniFrac distance between UC-HMA mice given UC or healthy VLPs. Related to Fig. 6. Supplementary Figure S11. PCoA on weighted UniFrac distance between UC-HMA mice given UC VLPs or heat-killed UC VLPs. Related to Fig. 6. Supplementary Figure S12. Relative abundance of temperate phages. Related to Fig. 6. Supplementary Figure S13. DSS does not induce UC gut bacterial prophages in vitro. Related to Fig. 6. Supplementary Figure S14. Human microbiota protects mice from experimental colitis. Related to Fig. 7. Supplementary Table S2. PERMANOVA and effect size of weighted UniFrac distances of bacterial communities between UC-HMA mice given a single dose of healthy VLPs or UC VLPs. Related to Fig. 3. Supplementary Table S3. PERMANOVA and effect size of weighted UniFrac distances of bacterial communities between healthy-HMA mic [file 40168_2022_1275_MOESM1_ESM.docx]

**SUPPLEMENTARY FIGURES**

**
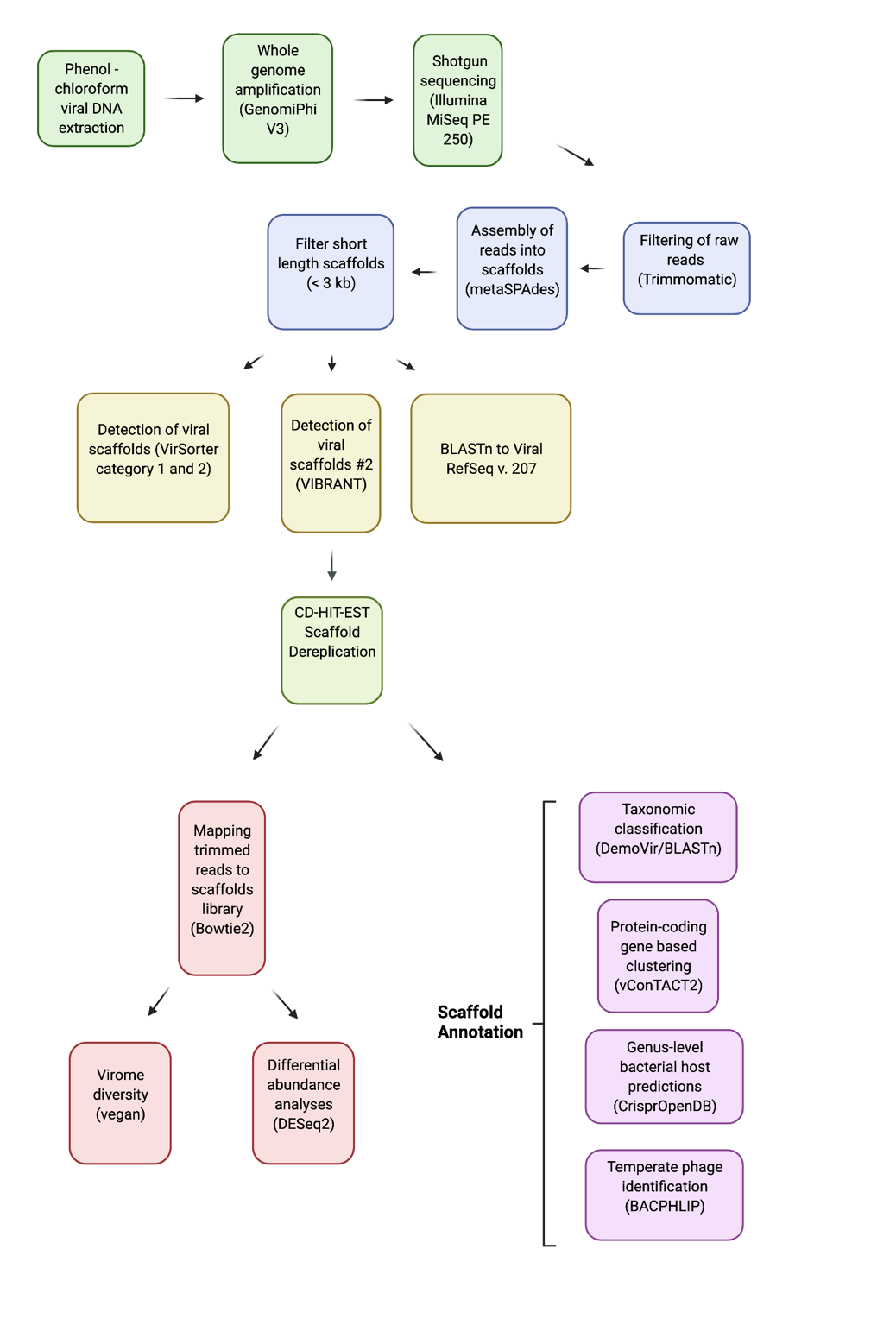
**

**Supplementary Figure S1.** **Virome analyses on inoculum and mouse VLPs**. **Related to Figure 2 and Figure 5. (**A) Viral metagenomic analyses was performed on fecal pellets from UC-HMA mice and human pooled UC or healthy VLP inoculums. Viral scaffolds were detected from mice and human samples and pooled to form a non-redundant scaffolds library.

**Supplementary Figure S2. Experimental colitis severity of UC-HMA and healthy-HMA mice given a single dose of healthy or UC VLPs.** **Related to Figure 3.** Data shown is from experiment ‘A’. (A) Mean absolute number and frequency of inflammatory monocytes (CD11b+Ly6C+Ly6G-) isolated from the colon at day 10 post-DSS administration. (B) Mean absolute number and frequency of neutrophils (CD11b+Ly6C-Ly6G+) isolated from the colon at day 10 post-DSS administration. (C) Mean Inflammatory cytokine production from colon tissue explants at day 10 post-DSS administration. Data were analyzed by one-way ANOVA, using Tukey’s multiple comparison test. Dots represent individual mice. Hbac + healthy/UC VLPs, n = 4 mice per group; UCbac + healthy/UC VLPs, n=6 mice per group. Hbac, healthy-HMA mice; UCbac, UC-HMA mice.

**Supplementary Figure S3. Viral and bacterial abundance in UC-HMA mice given UC or healthy VLPs**. **Related to Figure 4.** Data shown is from experiment ‘B’ (trial #2). (A) Viral abundance was determined from mouse fecal pellets using epifluorescence microscopy and compared to (B) bacterial abundances obtained by flow cytometry after staining with SybrGREEN I to obtain (C) VBRs. (D) Mean total viral abundance and mean total VBR post-VLP gavage were compared between treatment groups after the first dose of VLPs or PBS was given to mice. (A-C) Significance was assessed using a repeated measures two-way ANOVA and Dunnett’s multiple comparisons test (*p $\leq$ 0.05, **p$\leq$ 0.01) and using a Geisser-Greenhouse correction. Red and blue asterisks indicate significant differences between the PBS control and HMA mice given UC VLPs and healthy VLPs, respectively. Dots represent abundance or VBR of pooled mouse fecal samples at a single sampling point. At each sampling point, mouse fecal samples in each cage were pooled from 2 mice (n=3 cages per group, 6 mice per group). Error bars, SE. UC bac, UC-HMA mice.

**Supplementary Figure S4. Viral and bacterial abundance in UC-HMA mice given intact or heat-killed UC VLPs**. **Related to Figure 4.** Data shown from experiment ‘C’. (A) Viral abundance was determined from mouse fecal pellets using epifluorescence microscopy and compared to (B) bacterial abundances obtained by flow cytometry after staining with SybrGREEN I to obtain (C) VBRs. (D) Mean total viral abundance and mean total VBR post-VLP gavage were compared between treatment groups after the first dose of VLPs or heat-killed VLPs was given to mice. (A-C) Significance was assessed using a repeated measures two-way ANOVA and Dunnett’s multiple comparisons test (*p $\leq$ 0.05, ***p$\leq$ 0.001) and using a Geisser-Greenhouse correction. Gold and red asterisks indicate significant differences between heat-killed controls and HMA mice given UC VLPs (-DSS) or UC VLPs (+DSS) respectively. Dots represent abundance or VBR of pooled mouse fecal samples at a single sampling point. At each sampling point, mouse fecal samples in each cage were pooled from 2 mice (n=3 cages per group, 6 mice per group). Error bars, SE. UC bac, UC-HMA mice.

**Supplementary Figure S5. Bacterial activity in HMA mice given UC or healthy VLPs. Related to Figure 4.** Data shown is from (B) experiment ‘B’ (trial #1), (C) experiment ‘B’ (trial #2) and (D) experiment ‘C’. Bacterial communities were extracted from mouse fecal pellets under anaerobic conditions. To determine the proportion of active bacterial cells, fecal bacterial communities were stained with SybrGreen. (A) Gating strategy for mouse fecal bacteria to determine bacterial activity. Cells with high green fluorescence were determined high nucleic acid (HNA, active). (B-C) Red and blue asterisks indicate significant differences between the PBS control and HMA mice given UC VLPs or healthy VLPs respectively. (D) Gold and red asterisks indicate significant differences between heat-killed controls and HMA mice given UC VLPs (-DSS) and UC VLPs (+DSS) respectively. Significance at each sampling point was assessed using Dunnett’s multiple comparisons test (***p $\leq$ 0.001) and using a Geisser-Greenhouse correction. At each sampling point, mouse fecal samples in each cage were pooled from 2 mice (n=3 cages per group, 6 mice per group). Dots represent active or damaged bacterial cells from pooled mouse fecal samples at a single sampling point. Error bars, SE. UC bac, UC HMA-mice.

**Supplementary Figure S6. Bacterial damage in HMA mice given UC or healthy VLPs. Related to Figure 4.** Data shown is from (B) experiment B (trial #1), (C) experiment B (trial# 2) and (D) experiment ‘C’. Bacterial communities were extracted from mouse fecal pellets under anaerobic conditions. To determine the proportion of damaged bacterial cells, fecal bacterial communities were stained with PI. (A) Gating strategy for mouse fecal bacteria to determine bacterial cell damage. Cells with high red fluorescence were determined damaged. (B-C) Red and blue asterisks indicate significant differences between the PBS control and HMA mice given UC VLPs or healthy VLPs respectively. (D) Gold and red asterisks indicate significant differences between heat-killed controls and HMA mice given UC VLPs (-DSS) and UC VLPs (+DSS) respectively. Significance at each sampling point was assessed using Dunnett’s multiple comparisons test (*p $\leq$ 0.05, **p $\leq$ 0.001) and using a Geisser-Greenhouse correction. At each sampling point, mouse fecal samples in each cage were pooled from 2 mice (n=3 cages per group, 6 mice per group). Dots represent active or damaged bacterial cells from pooled mouse fecal samples at a single sampling point. Error bars, SE. UC bac, UC HMA-mice.

**Supplementary Figure S7. Richness of viral scaffolds and viral clusters in UC-HMA mice given healthy or UC VLPs. Related to Figure 5.** Data shown is from experiment ‘B’ (trial #1). **(**A-B) Richness of viral scaffolds. (C-D) Richness of vConTACT2 viral clusters (including outliers and singletons). (A, C) Mean viral richness per sampling point ± SE. (B,D) Mean viral richness ± SE grouping all sampling points post-VLP gavage. UCbac, UC-HMA mice.

**Supplementary Figure S8. Jaccard distance to pooled healthy VLP and UC VLP inoculums over time in UC-HMA mice given UC or healthy VLPs. Related to Figure 5.** Data shown is from experiment ‘B’ (trial #1). (A, C) Mean Jaccard distance over time to the (A) pooled healthy VLP inoculum in mice given HP VLPs and (C) pooled UC VLP inoculum in mice given UC VLPs. (B,D) Mean Jaccard distance per cage pre-VLP gavage and post-VLP gavage. Each dot represents the mean Jaccard distance to the VLP inoculum in a single cage. Significance was assessed using the Wilcoxin test.

**
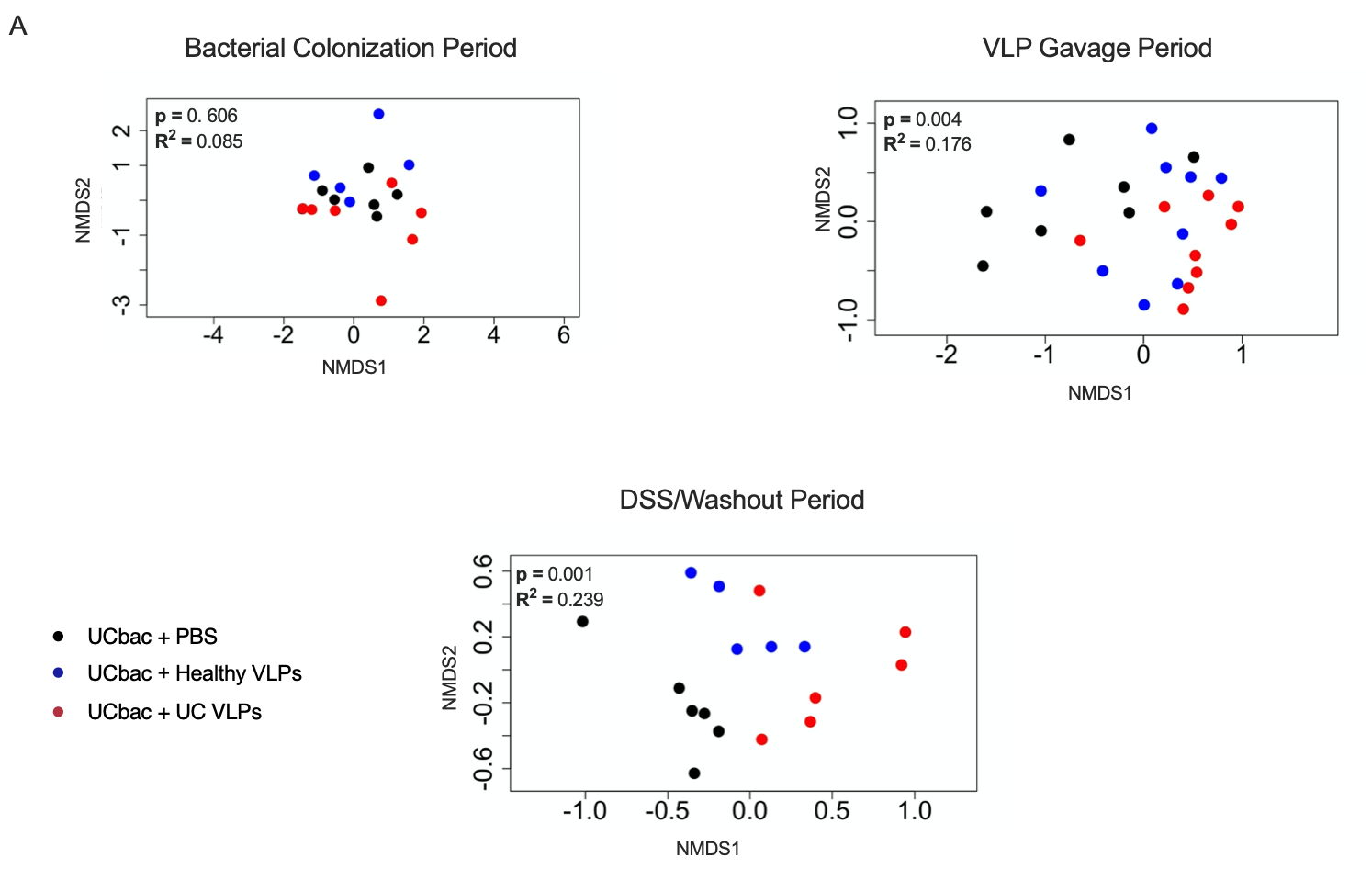
**

**Supplementary Figure S9. NMDS on Bray-Curtis dissimilarity of viral clusters between UC-HMA mice given UC or healthy VLPs. Related to Figure 5.** Data shown is from experiment ‘B’ (trial #1). (A) NMDS of Bray-Curtis dissimilarity of viral clusters between HMA mice given healthy VLPs, UC VLPs or PBS during the bacterial colonization period, VLP gavage period or DSS/washout period. Significant differences in Bray-Curtis dissimilarity were assessed in each time period using adonis PERMANOVA (p $\leq$ 0.05). Dots represent pooled mouse fecal samples at a single sampling point. Samples from all sampling points of the longitudinal study were included in the NMDS and comparative analyses. NMDS stress: bacterial colonization period (stress = 0.145), VLP gavage period (stress = 0.177), DSS/washout (stress = 0.184). UCbac, UC-HMA mice.

**
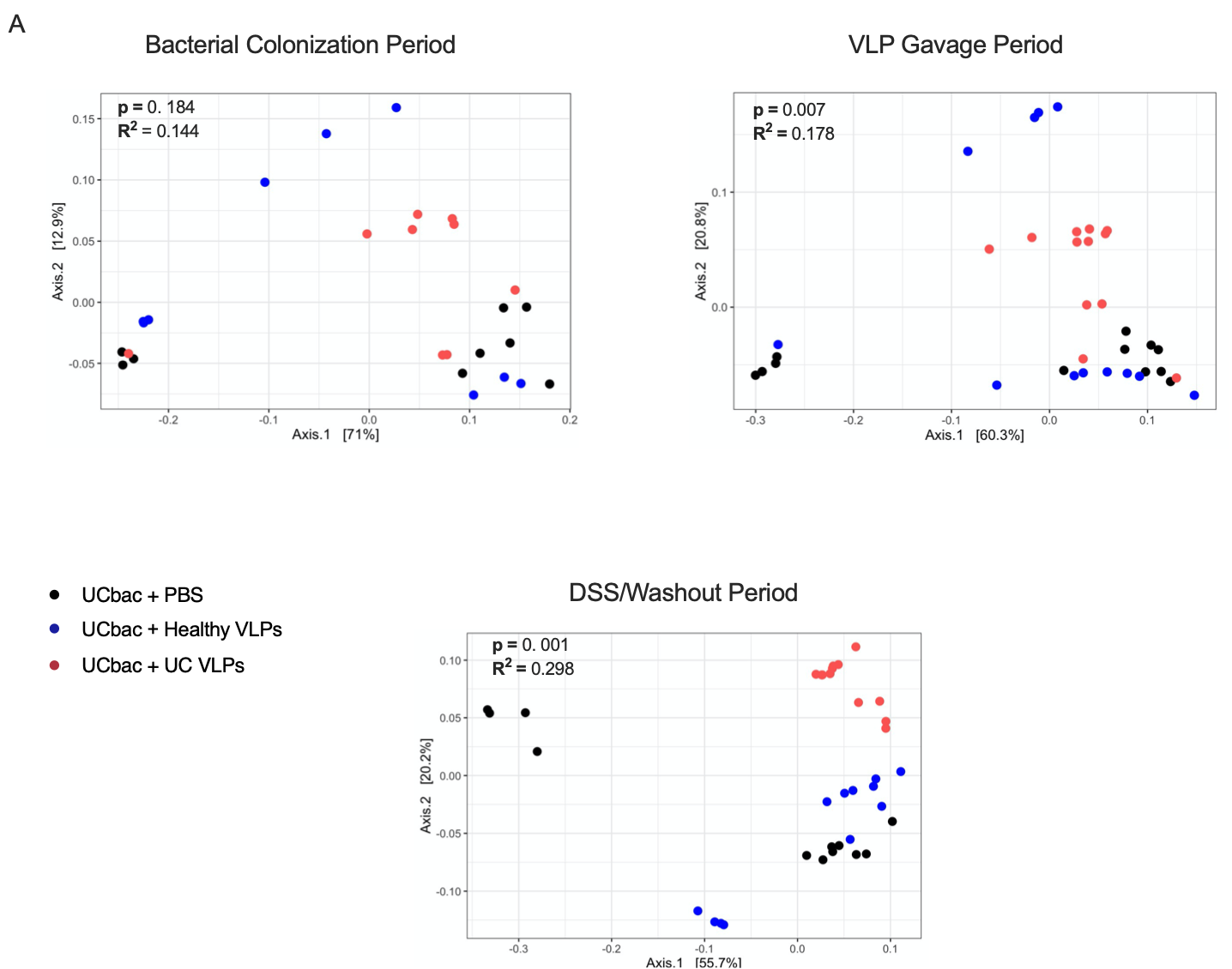
**

**Supplementary Figure S10. PCoA on weighted UniFrac distance between UC-HMA mice given UC or healthy VLPs. Related to Figure 6.** Data shown is from experiment ‘B’ (trial #2). PCoA on weighted UniFrac distance between HMA mice during the bacterial colonization period, VLP gavage period and DSS/washout period. Significant differences between weighted UniFrac distances were assessed using PERMANOVA (p $\leq$ 0.05) (bottom). Samples from all sampling points were included in the PCoA and comparative analyses. Mouse fecal samples in each cage were pooled from 2 mice (n=3 cages, 6 mice per treatment group). Dots represent pooled mouse fecal samples at a single sampling point. UCbac, UC-HMA mice.

**Supplementary Figure S11. PCoA on weighted UniFrac distance between UC-HMA mice given UC VLPs or heat-killed UC VLPs. Related to Figure 6.** Data shown is from experiment ‘C’. PCoA on weighted UniFrac distance between HMA mice during the bacterial colonization period, VLP gavage period and DSS/washout period. Significant differences between weighted UniFrac distances were assessed using PERMANOVA (p $\leq$ 0.05) (bottom). Samples from all sampling points were included in the PCoA and comparative analyses. Mouse fecal samples in each cage were pooled from 2 mice (n=3 cages, 6 mice per treatment group). Dots represent pooled mouse fecal samples at a single sampling point. UCbac, UC-HMA mice.

**Supplementary Figure S12. Relative abundance of temperate phages. Related to Figure 6.** Data shown is from experiment ‘B’ (trial #1). Mean relative abundance of temperate phages pre-DSS and post DSS in UC-HMA mice given (A) PBS (B) healthy VLPs, or (C) UC VLPs. Temperate phages were classified using BACPHLIP Mouse fecal samples in each cage were pooled from 2 mice (n=3 cages, 6 mice per treatment group). UCbac, UC-HMA mice.

**Supplementary Figure S13. DSS does not induce UC gut bacterial prophages *in vitro.* Related Figure 6. (**A) Mean VLP abundance from the supernatant of bacterial cultures from pooled UC fecal samples grown *in vitro*. Bacteria were grown anaerobically in triplicate at 37°C in BHI media supplemented with hemin (5 μg/mL) and vitamin K (1 μg/mL). DSS or H_2_O were added to cultures at early exponential phase (0.25-3 OD_600_) and VLP supernatant was sampled for enumeration at stationary phase after 14 hr of growth.

**
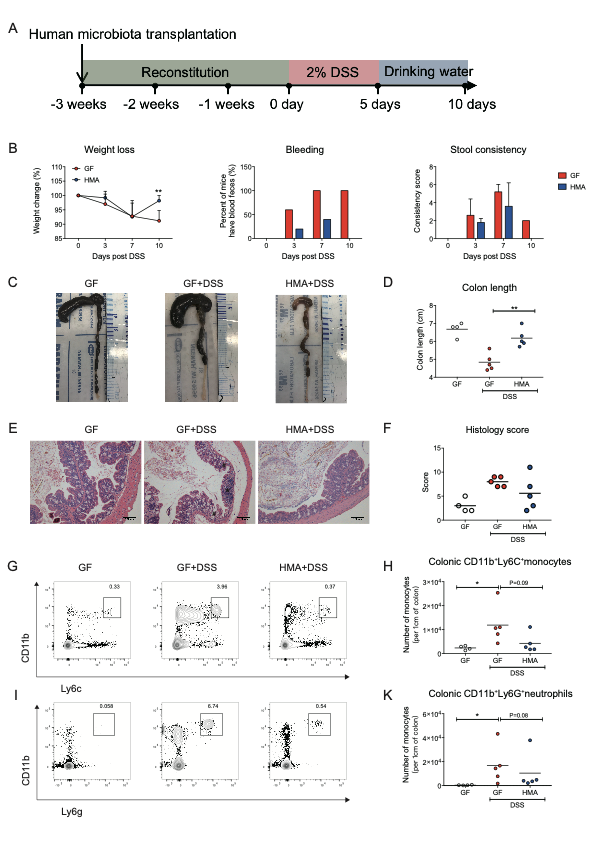
**

**Supplementary Figure S14. Human microbiota protects mice from experimental colitis.** **Related to Figure 7.** (A) Experimental design of DSS-colitis on HMA mice. (B) Changes of clinical disease activity index (DAI) including mean weight loss (left), mean GI tract bleeding (middle), and mean stool consistency (right) following DSS treatment. n=5 mice for each group. (C) Gross picture of colons at day 10 post DSS administration. (D) Mean length of colon of Control GF mice without DSS (white), GF mice (red) and HMA mice (blue) at day 10 post DSS administration. (E) Representative H&E staining of paraffin-embedded colon tissue at day 10 post DSS administration (scale bars are 100μm). Asterisk (*) indicates area of cellular infiltration; Number sign (#) indicates area of distortion of crypt architecture. (F) Mean histopathology of the effect of human gut microbiota colonization on DSS-induced colon damage in mice at day 10 post DSS administration. (G) Representative contour plots of colon monocytes defined as Viability-CD45+Ly6G-CD11b+Ly6C+ cells. (H) Mean total cell numbers of colon monocytes at day 10 post DSS administration. (I) Representative contour plots of colon neutrophils defined as Viability-CD45+CD11b+Ly6G+ cells. (K) Mean total cell numbers of colon neutrophils at day 10 post DSS administration. B-K. Data shown from one experiment. Each dot represents an individual mouse. Data were analyzed by two-way ANOVA with Bonferroni for multiple comparisons for panel B; Data were analyzed using an two-tailed unpaired parametric t test (*p < 0.05, **p < 0.01, ***p < 0.001) for panels D, H, K. Error bars represent the SD.

**SUPPLEMENTARY TABLES**

| PERMANOVA on Weighed UniFrac Distances: UCB + UC VLPs vs. UCB + Healthy VLPs | | | |
| --- | --- | --- | --- |
|  | **Day** | **p-value** | **R^2^** |
| **VLP Gavage Period** | 22 | 0.4 | 0.165 |
|  | 29 | 0.4 | 0.195 |
| **DSS/Washout Period** | 30 | 0.2 | 0.416 |
|  | 34 | 0.2 | 0.286 |
|  | 37 | 0.2 | 0.323 |
|  | 41 | 0.7 | 0.107 |

**Supplementary Table S2. PERMANOVA and effect size of weighted UniFrac distances of bacterial communities between UC-HMA mice given a single dose of healthy VLPs or UC VLPs. Related to Figure 3.** Data shown is from experiment ‘A’. Mouse fecal samples in each cage were pooled from 1 or 2 mice (n=3 cages per treatment group, 1 or 2 mice per cage). Hbac, healthy-HMA mice; UCbac, UC-HMA mice.

| PERMANOVA on Weighed UniFrac Distances: HB + UC VLPs vs. HB + Healthy VLPs | | | |
| --- | --- | --- | --- |
|  | **Day** | **p-value** | **R^2^** |
| **VLP Gavage Period** | 22 | 0.6 | 0.164 |
|  | 29 | 0.7 | 0.124 |
| **DSS/Washout Period** | 30 | 0.3 | 0.260 |
|  | 34 | 0.6 | 0.180 |
|  | 37 | 0.4 | 0.240 |
|  | 41 | 0.1 | 0.535 |

**Supplementary Table S3. PERMANOVA and effect size of weighted UniFrac distances of bacterial communities between healthy-HMA mice given a single dose of healthy VLPs or UC VLPs at each sampling point. Related to Figure 3.** Data shown is from experiment ‘A’. Mouse fecal samples in each cage were pooled from 1 or 2 mice (n=3 cages per treatment group, 1 or 2 mice per cage). Hbac, healthy-HMA mice; UCbac, UC-HMA mice.

| PERMANOVA on Weighed UniFrac Distances: UCB vs. HB | | | |
| --- | --- | --- | --- |
|  | **Day** | **p-value** | **R^2^** |
| **Bacterial Colonization Period** | 7 | 0.025 | 0.721 |
|  | 14 | 0.028 | 0.653 |
|  | 21 | 0.04 | 0.580 |
| **VLP Gavage Period** | 22 | 0.003 | 0.408 |
|  | 29 | 0.006 | 0.448 |
| **DSS/Washout Period** | 30 | 0.014 | 0.400 |
|  | 34 | 0.042 | 0.237 |
|  | 37 | 0.004 | 0.397 |
|  | 41 | 0.056 | 0.208 |

**Supplementary Table S4. PERMANOVA and effect size of weighted UniFrac distances of bacterial communities between UC-HMA mice and healthy-HMA mice at each sampling point. Related to Figure 3.**  Data shown is from experiment ‘A’. Mouse fecal samples in each cage were pooled from 1 or 2 mice (n=3 cages per treatment).

| **PERMANOVA on Bray-Curtis dissimilarity of viral scaffolds and viral clusters** | | | | | |
| --- | --- | --- | --- | --- | --- |
|  | **Day** | **p-value (viral scaffolds)** | **R^2^ (viral scaffolds)** | **p-value (viral clusters)** | **R^2^ (viral clusters)** |
| **Bacterial Colonization Period** | 7 | Not enough samples in each group | | |  |
|  | 14 | Not enough samples in each group | | |  |
|  | 21 | 0.053 | 0.315 | 0.064 | 0.317 |
| **VLP Gavage Period** | 22 | 0.049 | 0.361 | 0.042 | 0.363 |
|  | 26 | 0.015 | 0.358 | 0.023 | 0.346 |
|  | 29 | Not enough samples in each group | | |  |
| **DSS/ Washout Period** | 33 | Not enough samples in each group | | |  |
|  | 35 | Not enough samples in each group | | |  |
|  | 37 | Not enough samples in each group | | |  |
|  | 39 | 0.027 | 0.316 | 0.04 | 0.305 |

**Supplementary Table S7. PERMANOVA and effect size of Bray-Curtis dissimilarity on viral scaffolds and viral clusters between UC-HMA mice given healthy and UC VLPs. Related to Figure 5.** Data shown is from experiment ‘B’ (trial #1). Significant differences in Bray-Curtis dissimilarity were assessed at each sampling point using adonis PERMANOVA (p $\leq$ 0.05). At some sampling points, comparisons could not be made since some samples were excluded for shotgun sequencing due to an insufficient DNA yield.

| **ANCOM II Differentially abundant bacterial species** | | | | | | | |
| --- | --- | --- | --- | --- | --- | --- | --- |
| **Bacterial Species** | **Pairwise Difference** | **Time Period Where Significant** | **W > 0.6** | **W > 0.7** | **W > 0.8** | **W > 0.9** |  |
| *Anaerotruncus* sp. (2) trial #1 | Decreased in UC VLP treatment | VLP gavage and DSS/ Washout | TRUE | FALSE | FALSE | FALSE |  |
| *Sellimonas* sp. (3) trial #1 | Increased in UC VLP treatment | DSS/Washout | TRUE | TRUE | FALSE | FALSE |  |
| *Eubacterium limosum* (2) trial #1 | Decreased in UC VLP treatment | DSS/Washout | TRUE | TRUE | TRUE | TRUE |  |
| [*Eubacterium*] *fissicatena* group sp. (3) trial #2 | Increased in healthy VLP treatment | VLP gavage and DSS/ Washout | TRUE | FALSE | FALSE | FALSE |  |
| *Enterococcus* sp. (1) trial #2 | Decreased in PBS control | DSS/Washout | TRUE | FALSE | FALSE | FALSE |  |
| [*Eubacterium*] *coprostanoligenes* sp. (2) trial #2 | Increased in PBS control treatment | DSS/Washout | TRUE | TRUE | TRUE | FALSE |  |
| *Butyricicoccus* sp. (2) trial #2 | Increased in healthy VLP treatment | DSS/Washout | TRUE | TRUE | TRUE | FALSE |  |
| *Negativibacillus* sp. (3) trial #2 | Increased in UC VLP treatment | DSS/Washout | TRUE | TRUE | TRUE | FALSE |  |
| *Ruminococcaceae* UCG-005 sp. (3) trial #2 | Increased in UC VLP treatment | DSS/Washout | TRUE | TRUE | TRUE | FALSE |  |
| Uncultured *Clostridium* sp. (2) trial #2 | Increased in healthy VLP treatment | DSS/Washout | TRUE | TRUE | FALSE | FALSE |  |
| *Anaerotruncus* sp. (3) trial #2 | Increased in UC VLP treatment | DSS/Washout | TRUE | FALSE | FALSE | FALSE |  |
| Eubacterium corp  UBA1819 sp. (3) trial #2 | Increased in UC VLP treatment | DSS/Washout | TRUE | TRUE | TRUE | TRUE |  |

**Supplementary Table S9. Differentially abundant bacterial species during in HMA mice given healthy VLPs, UC VLPs, or PBS.** **Related to Figure 6**. Data shown is from experiment ‘B’ (trial #1 and trial #2**)**. Taxonomy was assigned using the Qiime2 feature classifier and differentially abundant species were determined using analysis of the composition of microbes (ANCOM II). Taxa that were found to be differentially abundant during bacterial colonization were not included. Numbers in parentheses (1-3) correspond to likelihood that pairwise differences were due to phage treatment or isolator effect (see STAR methods for ranking criteria). "TRUE” indicates that the bacterial species was found to be differentially abundant at the given W cutoff. In each experiment, mouse fecal samples in each cage were pooled from 2 mice (n=3 cages per treatment group).

| **ANCOM II Differentially abundant bacterial species** | | | | | | |
| --- | --- | --- | --- | --- | --- | --- |
| **Bacterial Species** | **Pairwise Difference** | **Time Period Where Significant** | **W > 0.6** | **W > 0.7** | **W > 0.8** | **W > 0.9** |
| *Blautia hydrogenotrophica* (3) | Increased in heat-killed UC VLP treatment | VLP gavage and DSS/Washout | TRUE | FALSE | FALSE | FALSE |
| *Alistipes* sp. (2) | Decreased in heat-killed UC VLP treatment | DSS/Washout | TRUE | TRUE | FALSE | FALSE |
| *Escherichia*–*Shigella* sp. (1) | Decreased in heat-killed UC VLP treatment | DSS/Washout | TRUE | TRUE | TRUE | TRUE |
| [*Eubacterium*] *fissicatena* group sp. (3) | Increased in heat-killed UC VLP treatment | DSS/Washout | TRUE | TRUE | TRUE | TRUE |

**Supplementary Table S10. Differentially abundant bacterial species in HMA mice given UC VLPs (+/- DSS), or heat-killed UC VLPs. Related to Figure 6.** Data shown is from experiment ‘C’. Taxonomy was assigned using the Qiime2 feature classifier and differentially abundant species between treatment groups were determined using analysis of the composition of microbes (ANCOM II). Taxa that were found to be differentially abundant during the bacterial colonization period were not included. Numbers in parentheses (1-3) correspond to likelihood that pairwise differences were due to VLP treatment or isolator effect (see STAR methods for ranking criteria). "TRUE” indicates that the bacterial species was found to be differentially abundant at the given W cutoff. Mouse fecal samples in each cage were pooled from 2 mice (n=3 cages per treatment group).

| **PERMANOVA on Weighed UniFrac Distances: UC-HMA mice given UC VLPs. Healthy VLPs or PBS (Trial #1)** | | | |
| --- | --- | --- | --- |
|  | **Day** | **p-value** | **R^2^** |
| **Bacterial Colonization Period** | **7** | 0.346 | 0.261 |
|  | **14** | 0.18 | 0.320 |
|  | **21** | 0.662 | 0.209 |
| **VLP Gavage Period** | **22** | 0.125 | 0.456 |
|  | **26** | 0.861 | 0.134 |
|  | **29** | 0.603 | 0.200 |
| **DSS/Washout Period** | **33** | 0.591 | 0.219 |
|  | **35** | 0.051 | 0.396 |
|  | **37** | 0.466 | 0.253 |
|  | **39** | 0.008 | 0.577 |

**Supplementary Table S11. PERMANOVA and effect size of weighted UniFrac distance on bacterial communities between UC-HMA mice given healthy VLPs, UC VLPs or PBS at each sampling point. Related to Figure 5.** Data shown is from experiment ‘B’ (trial #1). Significant differences in weighted UniFrac distance were assessed at each sampling point using PERMANOVA (p $\leq$ 0.05).

| **PERMANOVA on Weighed UniFrac Distances: UC-HMA mice given UC VLPs, Healthy VLPs or PBS (Trial #2)** | | | |
| --- | --- | --- | --- |
|  | **Day** | **p-value** | **R^2^** |
| **Bacterial Colonization Period** | 7 | 0.655 | 0.128 |
|  | 14 | 0.769 | 0.137 |
|  | 20 | 0.805 | 0.140 |
| **VLP Gavage Period** | 21 | 0.871 | 0.133 |
|  | 23 | 0.548 | 0.228 |
|  | 27 | 0.409 | 0.269 |
|  | 29 | 0.573 | 0.223 |
| **DSS/Washout Period** | 33 | 0.424 | 0.246 |
|  | 35 | 0.151 | 0.327 |
|  | 37 | 0.122 | 0.360 |
|  | 39 | 0.203 | 0.326 |

**Supplementary Table S12. PERMANOVA and effect size of weighted UniFrac distance on bacterial communities between UC-HMA mice given healthy VLPs, UC VLPs or PBS at each sampling point. Related to Figure 5.** Data shown is from experiment ‘B’ (trial #2). Significant differences in weighted UniFrac distance were assessed at each sampling point using PERMANOVA (p $\leq$ 0.05).

| **PERMANOVA on Weighed UniFrac Distances: UC-HMA mice given UC VLPs or Heat-Killed UC VLPs** | | | |
| --- | --- | --- | --- |
|  | **Day** | **p-value** | **R^2^** |
| **Bacterial Colonization Period** | 7 | 0.575 | 0.158 |
|  | 14 | 0.33 | 0.270 |
|  | 20 | 0.613 | 0.170 |
| **VLP Gavage Period** | 21 | 0.463 | 0.224 |
|  | 23 | 0.513 | 0.216 |
|  | 27 | 0.903 | 0.125 |
|  | 29 | 0.679 | 0.132 |
| **DSS/Washout Period** | 33 | 0.458 | 0.231 |
|  | 35 | 0.123 | 0.479 |
|  | 37 | 0.005 | 0.625 |
|  | 39 | 0.431 | 0.266 |

**Supplementary Table S13. PERMANOVA and effect size of weighted UniFrac distance on bacterial communities between UC-HMA mice given a at each sampling point. Related to Figure 5.** Data shown is from experiment ‘C’. Significant differences in weighted UniFrac distance were assessed at each sampling point using PERMANOVA (p $\leq$ 0.05).
